# Comparative Transcriptome and Metabolome Profiling Revealed Molecular Cascade Events During the Enzymatic Browning of Potato Tubers After Cutting

**DOI:** 10.3390/plants14121817

**Published:** 2025-06-13

**Authors:** Li Wang, Jianwei Shan, Jitao Liu, Kang An, Kun Yang, Chengchen Li, Xiaobo Li, Xingyao Xiong

**Affiliations:** 1 Research Institute of Crops, Provincial Key Laboratory of Crops Genetic Improvement, Guangdong Academy of Agricultural Sciences, Guangzhou 510640, China; wangli20090617@163.com (L.W.); ankang@gdaas.cn (K.A.);; 2 College of Horticulture, Hunan Agricultural University, Changsha 410128, China

**Keywords:** transcriptome, metabolome, potato tubers, cut-wounding, enzymatic browning

## Abstract

Enzymatic browning is a major issue in potato processing, causing a decline in both nutritional value and quality. Although there are numerous studies on the mechanism of enzymatic browning of potato tubers, few relevant reports are available on the changes at the transcriptome level during enzymatic browning as well as on the differences in the browning process of potato tubers with differing degrees of enzymatic browning potential. To gain insights into the molecular mechanism of enzymatic browning after cutting, this study presents the transcriptional characterization of temporal molecular events during enzymatic browning of browning-resistant (BR) and browning-susceptible (BS) potato tubers. RNA-sequencing (RNA-seq) analysis detected 19,377 and 13,741 differentially expressed genes (DEGs) in BR and BS tubers, respectively, with similar function enrichment observed using Gene Ontology (GO) and Kyoto Encyclopedia of Genes and Genomes (KEGG) enrichment analyses. Up-regulated DEGs were significantly enriched in the pathways related to phenol and lipid biosynthesis, while the down-regulated DEGs were significantly enriched in the pathways related to programmed cell death. Significant redox-related pathways occurred earlier in BS tubers compared to the BR tubers. Further analysis revealed that the BS tubers had a stronger phenolic synthesis ability compared to the BR tubers. However, the BR tubers showed a stronger free radical scavenging ability compared to the BS tubers. The results of our study provide insights into the temporal molecular events that occur during the enzymatic browning of potato tubers after cutting and the potential molecular mechanisms for different degrees of enzymatic browning.

## 1. Introduction

Enzymatic browning is the most significant phenomenon that takes place in fruits and vegetables during harvesting and processing. It leads to unfavorable changes in the sensory and nutritional properties and decreases the market value of various agricultural products [1,2]. Enzymatic browning is a complex process typically triggered by mechanical damage [3]. It is currently believed that polyphenol oxidase (PPO) and peroxidase (POD) are the main enzymes responsible for the enzymatic browning of plant tissues [4,5]. PPO, an oxidoreductase enzyme with four atoms of copper as a prosthetic group, which can catalyze the oxidation of the functional OH group attached to the carbon atom of the benzene ring of monohydroxy phenols to o-dihydroxy phenols and the dehydrogenation of odihydroxy phenols to o-quinones [6]. These o-quinones are then polymerized with intracellular macromolecules (e.g., proteins and amino acids) forming black, brown, and red pigments that cause browning of plant tissues [7]. Normally, enzymes and substrates are compartmentalized in plant cells—phenolic compounds in vacuoles and PPO in plastids [8,9]. When mechanical damage disrupts cellular integrity, the spatial separation of PPO and phenolic substrates is compromised, enabling their oxidizing reaction, further producing enzymatic browning. During the processing of agricultural products, tissue damage is inevitable, which leads to the enzymatic browning of tissues. POD, mainly involved in lignification processes, can also form melanins as previously described [4]. Unlike PPO, POD catalyzes the oxidation of phenolic compounds in the presence of H_2_O_2_ as a catalyst [4]. Richard-Forget and Gauillard (1991) also verified that the presence of PPO and POD enhanced phenol degradation along with the formation of quinones due to the production of H_2_O_2_ [4]. Phenylalanine ammonia-lyase (PAL) is the first enzyme in the pathway of phenylpropanoid metabolism and it is responsible for the synthesis of phenolic compounds, which can be induced by different stress conditions [10]. Hence, PAL activity was proposed as a possible index to determine the browning potential of minimally processed lettuce cultivars [11]. Expression of the PAL gene is regulated by a diverse array of factors, such as injury, infection, environment, and the developmental stage of plants [10].

Potato (*Solanum tuberosum* L.) is the world’s third most consumed food crop (http://www.fao.org/ (accessed on 20 December 2024)) and a key food security crop in developing countries [12]. There has been an increase in the consumption and production of potatoes worldwide due to their wide adaptive range, ease of cultivation, and high nutritional value [13]. However, potato tubers are susceptible to enzymatic browning, which has been considered a significant problem leading to economic loss [6,14]. Due to its browning characteristics, production costs involved in the processing of potato tubers, including fresh-cut ones and whole powder, have increased. Although many studies attribute enzymatic browning in bruised tubers to phenolic enzymes oxidizing phenolic compounds, changes in transcriptional regulation remain poorly understood, especially the difference between susceptible- and resistant-browning cultivar. In this study, we used browning-susceptible (BS) and browning-resistant (BR) potato cultivar tubers to understand the transcriptional changes in response to cut-wounding. Further, differences in the transcriptional regulation of potato tissues between two potato cultivars with varying degrees of browning potential were also investigated. The results of this study would provide insights into the temporal molecular events during the enzymatic browning of potato tubers after cutting and the potential molecular mechanisms for differing degrees of enzymatic browning.

## 2. Results

### 2.1. Enzymatic Browning and Activities of Total Phenols, PPO, and POD in Potato Tubers

**Figure 1:**
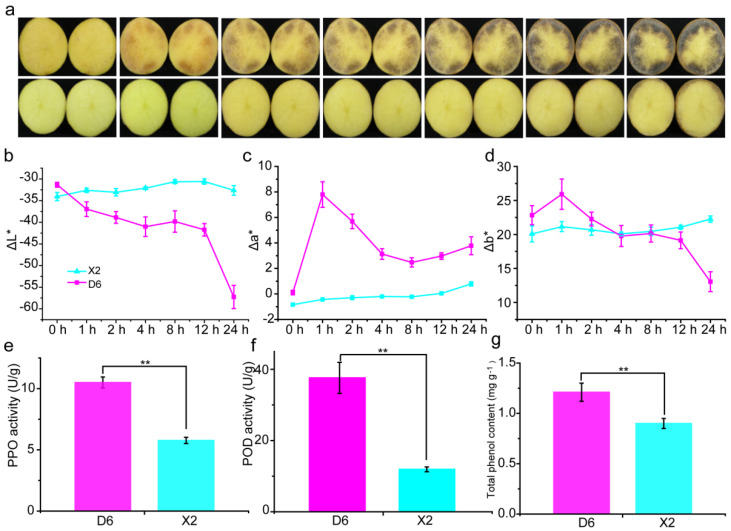
Enzymatic browning phenotype and related enzyme activity of potato tubers. (**a**) Enzymatic browning phenotype of potato tubers during 0–24 h after cutting. (**b**–**d**).Color change of potato tubers during 0–24 h after cutting. (**e**–**g**) PPO, POD, and total phenol content of potato tubers. D6, susceptible-browning cultivar; X2, resistant-browning cultivar. The mean value and standard error were obtained from three biological replicates, and the significance difference level *p* ≤ 0.01 (**).

Enzymatic browning of two different potato cultivar tubers, including a BR cultivar X2 and a BS cultivar D6, was investigated. The results showed that the D6 tubers suffered significant enzymatic browning at 1 h of cutting, and that the browning intensified with an increase in time. The X2 tubers showed browning at the tuber edge after 8 h of cutting, where both the ring medulla and the inner medulla displayed no significant browning until 24 h after cutting (Figure 1a). In D6 tubers, ΔL* continues to decrease over time, while Δa* and Δb* dramatically increase at 1 h after cutting and then decrease, which is consistent with phenotype of D6 tubers after cutting. Compared with D6 tubers, the ΔL*, Δa*, and Δb* of X2 tubers did not change significantly (Figure 1b–d). Furthermore, PPO and POD activities and total phenolic content were measured. The results showed that the D6 tubers had significantly higher levels of PPO and POD activities and total phenolic content compared to the X2 tubers (Figure 1e–g). These results indicated that the enzymatic browning characteristics of the two potato cultivars were different.

In our previous study, the expression of StuPPO genes was up-regulated at 0–24 h of cutting with prominent up-regulation observed at 4 h after cutting. Most StuPPO genes reached their highest expression levels at 12 and 24 h, respectively, in the D6 and X2 tubers after cutting [15]. Based on this, samples were collected at 0, 4, 12, and 24 h after cutting for the RNA-sequencing (RNA-seq) analysis in our study.

### 2.2. RNA-Seq and De Novo Assembly of Potato Transcriptome

A total of 458.4 million raw reads were generated (Table S1). After removing low-quality reads and adaptor sequences from the reads, a total of 557.68 and 452.54 million clean reads were detected across all samples of the two cultivars, which yielded 83.66 and 67.87 Gb nucleotides, respectively. GC content was 42.29–44.17% and the percentages of Q20 and Q30 were 96.3–97.55% and 90.54–93.23%, respectively. Clean reads were mapped to the potato reference genome using HISAT, and those that were successfully mapped to the reference genome were 85–87.66% and 69.27–83.46%, respectively, in the X2 and D6 samples (Table S2). Most of the total reads were uniquely mapped to the potato genome, whereas a small proportion (about 2.09–4.04% of the total mapped reads) were mapped to multiple locations in the potato genome. Pearson correlation coefficient between the three replications of each sample was more than 0.981 (Figure S1), indicating homogenous gene expression in the three replications of the samples. These results suggested that the sequencing output and its quality were sufficient to warrant further analysis. To confirm the accuracy and reliability of the transcriptome analysis data, 20 unigenes (Table S3) were randomly selected for quantitative real-time PCR (qRT-PCR) assays. By qRT-PCR, the trends observed in the expression of the unigenes were consistent with those observed in the RNA-seq data (Figure S2). Overall, these results suggested that the transcriptome analysis of our study using RNA-seq was reliable.

### 2.3. Differentially Expressed Genes (DEGs) of Potato Tubers at Different Time Points After Cutting

**Figure 2:**
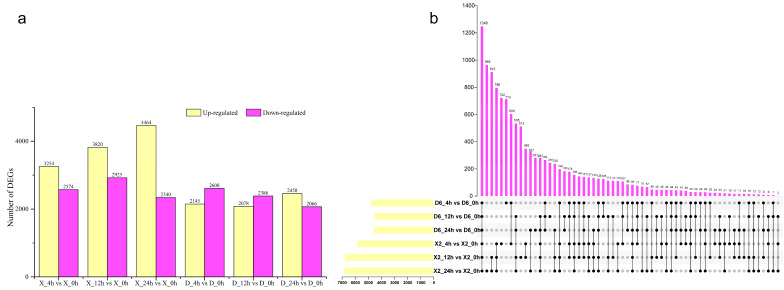
Statistical analysis of all identified differentially expressed genes (DEGs) in response to cut-wounding at different times. (**a**) Statistical chart of DEGs of transcriptome in response to cut-wounding at 4 h, 12 h, and 24 h after cutting; (**b**) Venn diagram of DEGs at different times after cut-wounding. D6, susceptible-browning cultivar; X2, resistant-browning cultivar.

Gene differential expression analysis based on |fold-change| ≥ 2 and *p*-value ≤ 0.05 identified 5828 (3254 up- and 2574 down-regulated), 6745 (3820 up- and 2925 down-regulated), 6804 (4464 up- and 2340 down-regulated) DEGs at 4, 12, and 24 h, respectively, compared to 0 h in the X2 potato tubers, while 4753 (2145 up- and 2608 down-regulated), 4464 (2078 up- and 2386 down-regulated), 4524 (2458 up- and 2066 down-regulated) DEGs were identified in the D6 tubers, respectively (Figure 2a). In general, the number of DEGs in the X2 tubers was more compared to the D6 tubers, and the proportion of up-regulated genes in the X2 tubers was significantly higher compared to that in the D6 tubers. This provided considerable differences between the two genotypes during enzymatic browning after cutting.

The overall GO categorization of DEGs at three different time points (4, 12, and 24 h) after cutting was similar in both X2 and D6 tubers. For example, 4318, 4930, and 5126 DEGs in X2 and 3639, 3305, and 3428 DEGs in D6 were split into two main categories, namely biological process and molecular function, which accounted for more than 87% of total GO terms (Table S4). Of the biological processes, the most dominant terms were the oxidation–reduction process, single-organism metabolic process, single-organism process, and the metabolic process. Of the molecular functions, the most dominant terms were oxidoreductase and catalytic activities (Figure S3). This indicated that the two different genotypes of potato tubers had a conserved transcriptional response during enzymatic browning after cutting.

To study DEGs’ general response to cut-wounding in potato tubers, 1248 DEGs overlapping in X2_4 h vs. X2_0 h, X2_12 h vs. X2_0 h, X2_24 h vs. X2_0 h, D6_4 h vs. D6_0 h, D6_12 h vs. D6_0 h, and D6_24 h vs. D6_0 h were identified (Figure 2b). It was found that the 1248 DEGs were involved in response to cut-wounding throughout 0–24 h of cutting in the two potato cultivars. GO analysis showed that the mainly enriched GO terms were redox-related functional terms (GO:0016491, GO:0016614, GO:0016616), oxidation-reduction process (GO:0055114), and single-organism process (GO:0044699), which included 244, 172, and 494 DEGs, respectively (Table 1).

**Figure 3:**
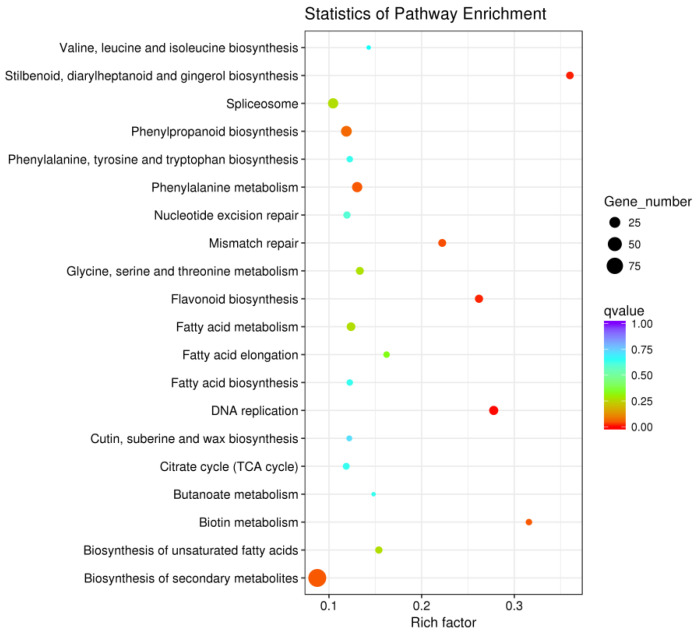
KEGG enrichment analysis of shared differentially expressed genes (DEGs) of X2 and D6 tubers after cutting.

Kyoto Encyclopedia of Genes and Genomes (KEGG) analysis showed that these DEGs mainly participated in the pathways of DNA replication, stilbenoid, diarylheptanoid, and gingerol biosynthesis, flavonoid biosynthesis, mismatch repair, biosynthesis of secondary metabolites, biotin metabolism, and phenylalanine metabolism (Figure 3 and Table S5). It can be speculated that these pathways are universal and play an important role in enzymatic browning after cutting. KEGG enrichment analysis provided an understanding of the mechanism of enzymatic browning after cutting in potato tubers.

**Figure 4:**
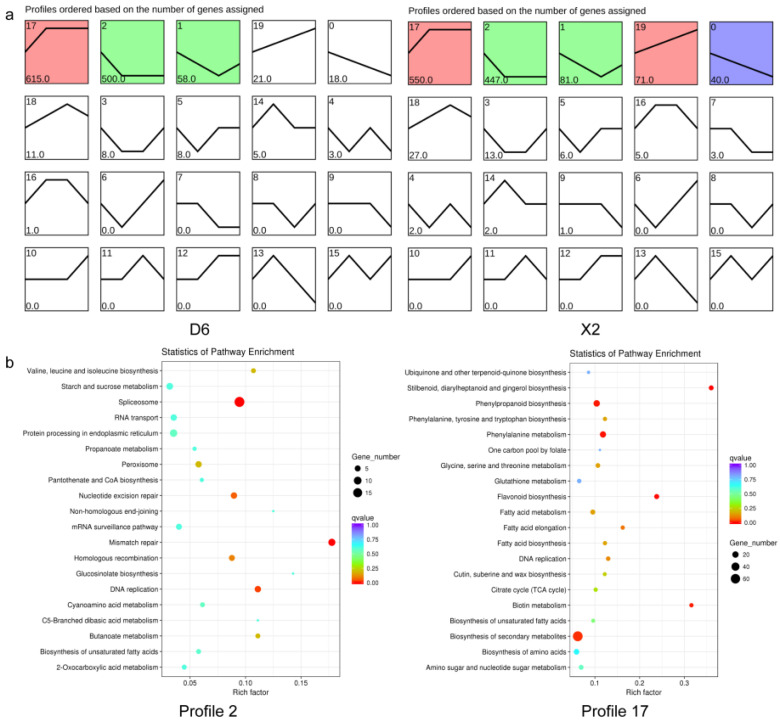
Gene expression pattern and KEGG analysis of 1248 overlapping DEGs. (**a**) Gene expression pattern analysis was performed by Short Time-series Expression Miner software (STEM version 1.3.13); (**b**) KEGG analysis of up- and down-regulated differentially expressed genes. D6, susceptible-browning cultivar; X2, resistant-browning cultivar.

Further, the expression trends of overlapping DEGs were examined. The results showed that profiles 17 and 2 had the greatest number of DEGs in both X2 and D6 tubers (Figure 4a). DEGs of profile 17 were up-regulated within 4 h and persisted throughout the 24 h experimental period. The sustained accumulation of this gene type suggested its biological significance during enzymatic browning after cutting in potato tubers. KEGG analysis was further performed for the DEGs of profiles 17 and 2, which showed that the DEGs of profile 17 significantly participated in the pathways of stilbenoid, diarylheptanoid, and gingerol biosynthesis, flavonoid biosynthesis, phenylalanine metabolism, phenylpropanoid biosynthesis, biotin metabolism, and the biosynthesis of secondary metabolites. The DEGs of profile 2, which were down-regulated, mainly participated in the pathways of the spliceosome, mismatch repair, and DNA replication (Figure 4b and Table S6). The up-regulated gene expression of profile 17 undoubtedly generated a suite of mechanisms required by the plant to contend with the stimulus, while the down-regulated genes of profile 2 suggested resource reallocation and remodeling of growth and development in response to the stimuli.

**Figure 5:**
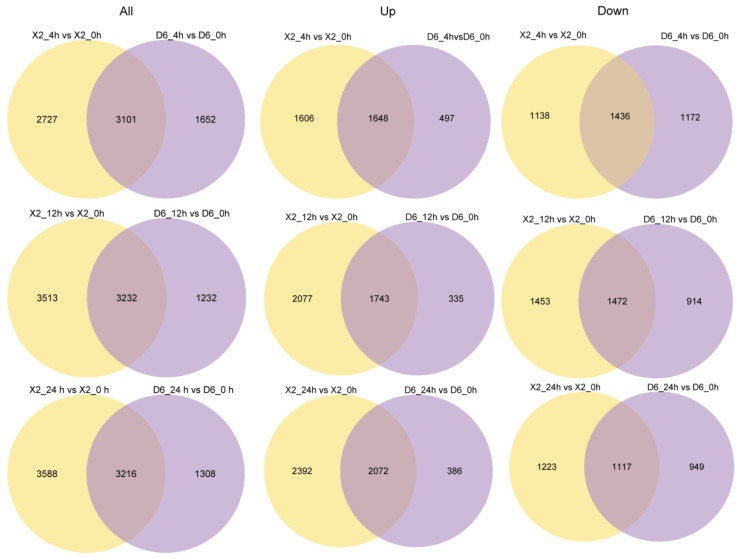
Venn diagram of differentially expressed genes (DEGs) of potato tubers at different times after cutting–wounding between X2 and D6 cultivars.

The overlapping genes of both potato cultivars explained the occurrence of a similar response in different genotypes. However, both potato tubers also had numerous genotype-specific DEGs, the analysis of which may reveal the possible causes of their differences in enzymatic browning characteristics. Hence, genotype-specific DEGs of X2 and D6 tubers were analyzed at different times of enzymatic browning after cutting (Figure 5). At 4 h of cutting, the X2 tubers showed 2727 specific DEGs, including 1606 up-regulated and 1138 down-regulated DEGs, while the D6 tubers showed 1652 specific DEGs, including 497 up-regulated and 1172 down-regulated DEGs. X2 tubers showed more up-regulated DEGs compared to the D6 tubers. GO analysis showed that the 1606 up-regulated genes of the X2 tubers were significantly enriched in protein phosphorylation, cellular protein, modification process, protein modification process, transferase activity, transferring hexosyl groups, transferase activity, transferring glycosyl groups, transferase activity. It also showed that the 1138 down-regulated DEGs of the X2 tubers were significantly enriched in ADP binding, transition metal ion binding, cation binding, and metal ion binding. The 497 up-regulated genes of the D6 tubers were significantly enriched in the oxidation-reduction process, single-organism metabolic process, and oxidoreductase activity, while its 1172 down-regulated DEGs were significantly enriched in ADP binding, transition metal ion binding, cation binding, and metal ion binding, similar to the X2 specific down-regulated DEGs (Table 2 and Table S7). These results indicated that the redox reaction was significant in the D6 tubers compared to the X2 tubers at the early stages of cutting, leading to an early occurrence of enzymatic browning in the D6 tubers.

At 12 h of cutting, X2 tubers reported 3513 genotype-specific DEGs, including 2077 up-regulated genes and 1453 down-regulated genes. The 2077 up-regulated genes were significantly enriched in catalytic activity, transferase activity, and oxidoreductase activity, while the 1453 down-regulated genes were significantly enriched in intracellular organelle part, organelle part, monovalent inorganic cation transport, and monovalent inorganic cation transmembrane transporter activity. D6 tubers reported 1232 genotype-specific DEGs, including 335 up-regulated genes and 914 down-regulated genes. The 335 up-regulated genes were significantly enriched in various metabolic processes, including various metabolic processes, gene expression, cytoplasmic part, intracellular organelle, and organelle cytoplasm. The 914 down-regulated genes were significantly enriched in cation binding, metal ion binding, ion binding, regulation of RNA biosynthetic process, regulation of RNA metabolic process, transcription, DNA-templated, regulation of transcription, and regulation of nucleic acid template transcription.

At 24 h of cutting, the X2 tubers reported 3588 genotype-specific DEGs, including 2392 up-regulated genes and 1223 down-regulated genes. The 2392 up-regulated genes were significantly enriched in catalytic activity, single-organism metabolic process, oxidation-reduction process, oxidoreductase activity, transferase activity, and metabolic process, while the 1223 down-regulated genes were significantly enriched in cation binding, metal ion binding, and nucleic acid binding. D6 tubers reported 1308 genotype-specific DEGs, including 386 up-regulated genes and 949 down-regulated genes. The 386 up-regulated genes were significantly enriched in oxidoreductase activity, oxidation-reduction process, catalytic activity, metabolic process, single-organism metabolic process, and single-organism process, while the 949 down-regulated genes were significantly enriched in ion binding and the regulation of different processes.

Notably, more up-regulated genes were detected in the X2 tubers, while more down-regulated genes were detected in the D6 tubers at three different time points. This indicated that the two different genotypes had different characteristics of enzymatic browning after cutting.

### 2.4. Critical Metabolic Pathways and Genes Related to Enzymatic Browning

#### 2.4.1. Phenylpropanoid Biosynthesis

The phenylpropanoid biosynthesis pathway was significantly enriched at all the time points during enzymatic browning after cutting. It is the main pathway responsible for the synthesis of phenolic compounds and secondary metabolites, as well as for the production of substrates for the flavonoid biosynthesis pathway and synthesis of stilbenoid, diarylheptanoid, and gingerol. In the D6 tubers, 54, 65, and 59 genes at 4, 12, and 24 h of cutting showed significant changes in expression, respectively, with their numbers being highest at 12 h. In the case of the X2 tubers, 66, 79, and 88 genes at 4, 12, and 24 h of cutting showed significant changes in expression, respectively, with their numbers being highest at 24 h of cutting. These results indicated that the phenylpropanoid pathway reached its maximum response faster in the D6 tubers compared to the X2 tubers during enzymatic browning. This was consistent with the previous speculation based on the expression profile of StuPPO genes, where the expression levels of StuPPO genes reached their highest at 12 and 24 h of cutting in BS and BR tubers, respectively [15].

**Figure 6:**
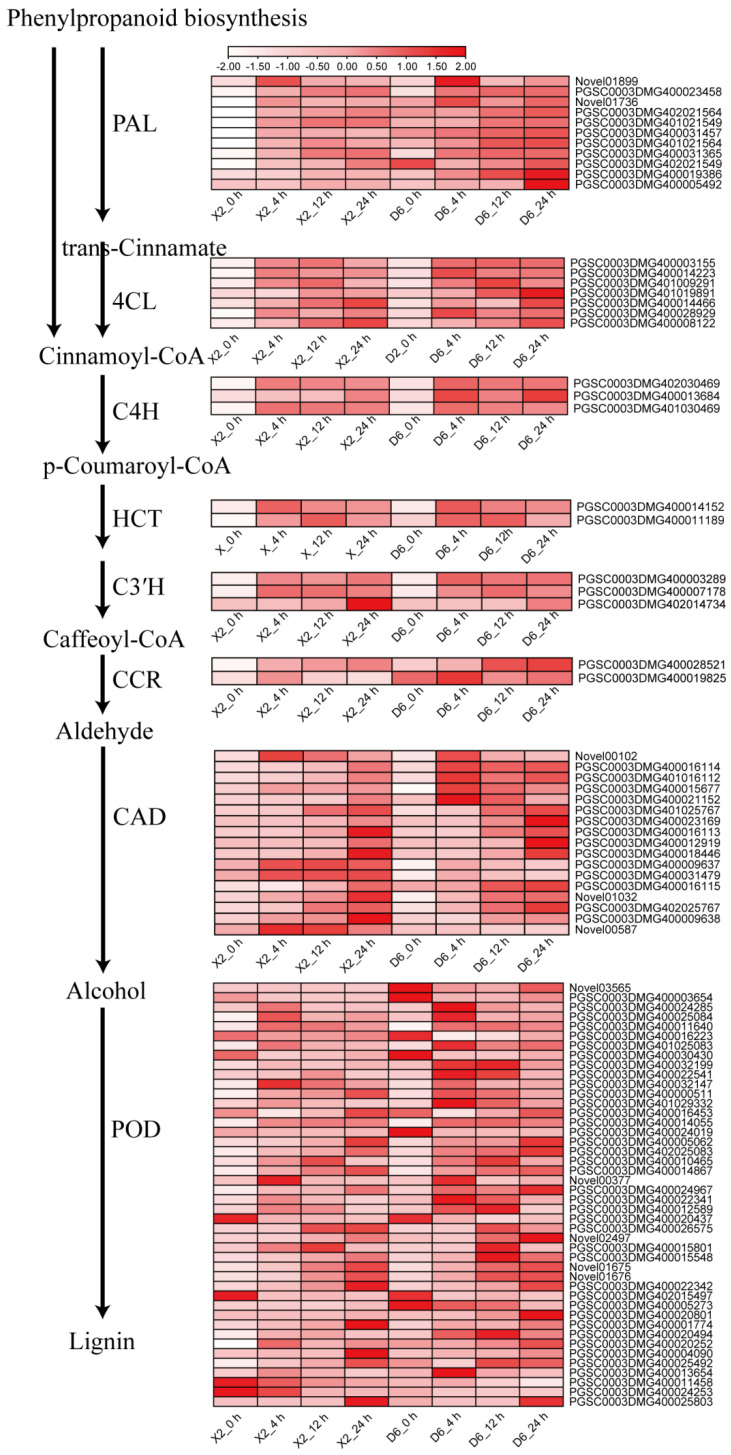
Expression patterns of differentially expressed genes (DEGs) in the pathway of phenylpropanoid biosynthesis response to cut-wounding in potato tubers at different times. Red represents a high transcript level and white represents a low transcript level. PAL: phenylalanine ammonia lyase; C4H: Cinnamate 4-hydroxylase; 4CL: 4-coumaroyl: CoA ligase; CCR: cinnamoyl CoA reductase; CAD: cinnamyl-alcohol dehydrogenase; POD: peroxidase; HCT: shikimate O-hydroxycinnamoyltransferase; C3′H: cytochrome P450 98A3-like; CCoAOMT: caffeoyl-CoA O-methyltransferase.

DEGs of the phenylpropanoid biosynthesis pathway include the genes encoding PAL, cinnamic acid 4-hydroxylase (C4H), 4-coumarate:CoA ligase (4CL), cinnamoyl-CoA reductase (CCR), shikimate O-hydroxycinnamoyltransferase (HCT), 5-O-(4-coumaroyl)-D-quinate 3′-monooxygenase (C3′H), cinnamyl-alcohol dehydrogenase (CAD), and POD. PAL is an enzyme that connects primary and secondary metabolism and catalyzes the first step in phenylpropanoid biosynthesis. It is also a key enzyme and a speed-limiting enzyme in the pathway of phenylpropanoid biosynthesis along with its participation in the synthesis of lignin, coumarate, and flavonoids. In this study, nine PAL genes were detected. Two, one, and two PAL genes were significantly up-regulated in the D6 tubers after cutting, respectively. In the X2 tubers, six genes were up-regulated at all three time points after cutting (Figure 6 and Table S8). It is worth noting that the expression levels of almost all the genes at the same time points after cutting were higher in the D6 tubers compared to the X2 tubers, despite a few showing no significant change in the D6 tubers. This indicated that the PAL genes had higher expression levels in the D6 tubers compared to the X2 tubers, which correlated with the higher total phenolic content observed in the D6 tubers (Figure 1).

4CL, C4H, HCT, C3′H, caffeoyl-CoA O-methyltransferase (CCoAOMT), and POD are the key enzymes involved in lignin synthesis. In this study, most of the 4CL, C4H, HCT, C3′H, and CCR genes were up-regulated in response to cut-wounding in both cultivar tubers with similar change trends. POD is the last key enzyme involved in lignin synthesis, which can also catalyze the enzymatic browning of plant tissues. Forty-three POD genes were detected in this study. Among them, 43 and 38 genes showed significant changes in the X2 and D6 tubers, respectively, and about 70.4% and 75% of these genes were up-regulated in response to cut-wounding in the X2 and D6 tubers, respectively. Similarly, the expression levels of most genes were higher in the D6 tubers compared to the X2 tubers at the same time points after cutting.

#### 2.4.2. Antioxidant Enzyme-Related Genes

**Figure 7:**
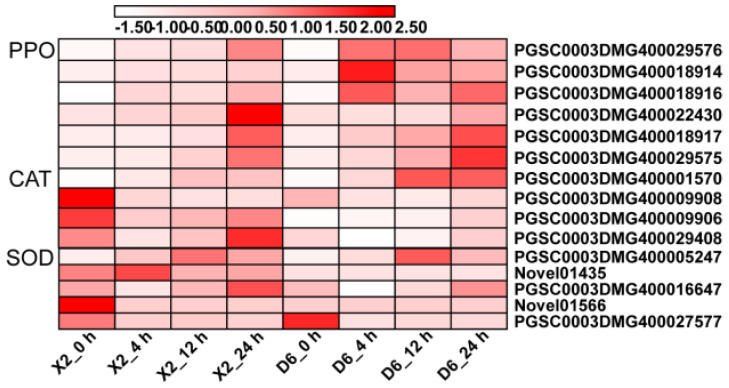
Expression patterns of DEGs-related antioxidant activity of potato tubers. Red represents a high transcript level and white represents a low transcript level. PPO: polyphenol oxidase; CAT: catalase; SOD: superoxide dismutase.

PPO is the main enzyme responsible for the enzymatic browning of plant tissues. In this study, six PPO gene transcripts were detected in potato tubers. A heat map of gene expression showed that the expression of the PPO genes increased continuously during enzymatic browning (Figure 7 and Table S9). Comparing the two cultivars, transcript levels of the PPO genes except for PGSC0003DMG400022430 in the D6 tubers were higher compared to the X2 tubers. Thus, it was proved that the PPO genes in BS tubers had higher expression levels, which correlated with their enzyme activities.

Catalase (CAT, EC:1.11.1.7) is a common redox enzyme in plants that eliminates H_2_O_2_ from plant tissues and offers protection from H_2_O_2_ toxicity. In our study, four CAT gene transcripts were detected. The expression levels of these genes were up-regulated at 0–24 h of cutting. Except for gene PGSC0003DMG400001570, the expression levels of the other three genes in the X2 tubers were higher compared to those in the D6 tubers. This demonstrated that the BR tubers may possess a stronger ability to scavenge H_2_O_2_.

Five superoxide dismutase (SOD) genes, whose expression levels were higher in the X2 tubers compared to the D6 tubers, were detected.

### 2.5. Redox-Related Enzyme Activity and H_2_O_2_ Content at Different Time After Cutting

**Figure 8:**
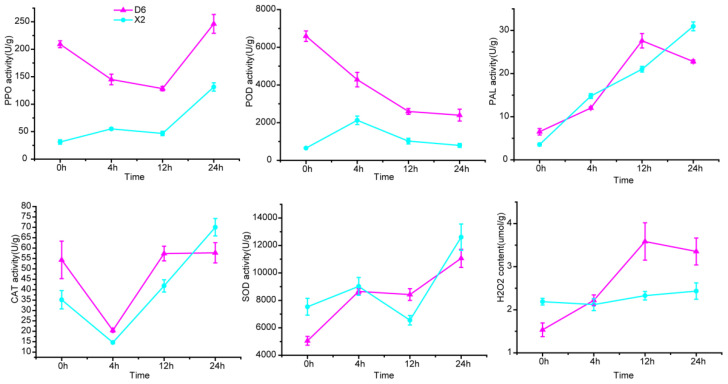
The enzymy activity and content of H_2_O_2_. The mean value and standard error were obtained from three biological replicates.

We determined PPO, POD, PAL, CAT, SOD activity and H_2_O_2_ content at different times in potato tubers (Figure 8). As expected, PPO and POD activities were significantly higher than those of X2 during 0–24 h after cutting. In D6 tubers, PPO continued to decrease from 0 to 12 h after cutting, and then increased; while in X2 tubers, there is a dramatic increase after 12 h after cutting. PAL activity reached the highest level at 12 h in D6 tubers, and subsequently decreased, while it then continued to increase. CAT activity in both cultivars′ tubers showed a trend of first decreasing and then increasing. SOD showed a similar trend in both cultivars. H_2_O_2_ is required for POD to catalyze the oxidation of phenolic compounds. Our results showed that in intact tubers, D6 had a lower H_2_O_2_ content than X2, but it increased significantly over time in D6 tubers and was significantly higher than that in X2, while the content in X2 did not change significantly during this period.

### 2.6. Changes in DEG-Related Plant Hormone Signal Transduction During Enzymatic Browning After Cutting

DEGs related to plant hormone signal transduction, including auxins (AUXs), cytokinins (CTKs), gibberellins (GAs), abscisic acid (ABA), ethylene (ET), brassinosteroid (BR), jasmonic acid (JA), and salicylic acid (SA) were 63, 37, and 46 at the three time points (4 h, 12 h, 24 h) during enzymatic browning after cutting in the D6 tubers, respectively. The highest number of DEGs was observed at 4 h, followed by a sharp decrease at 12 h, and an increase at 24 h. In the X2 tubers, 68, 67, and 51 DEGs were reported, respectively (Table S10). These results indicated that there were differences in plant hormone signal transduction-related genes during enzymatic browning between the D6 and X2 tubers.

**Figure 9:**
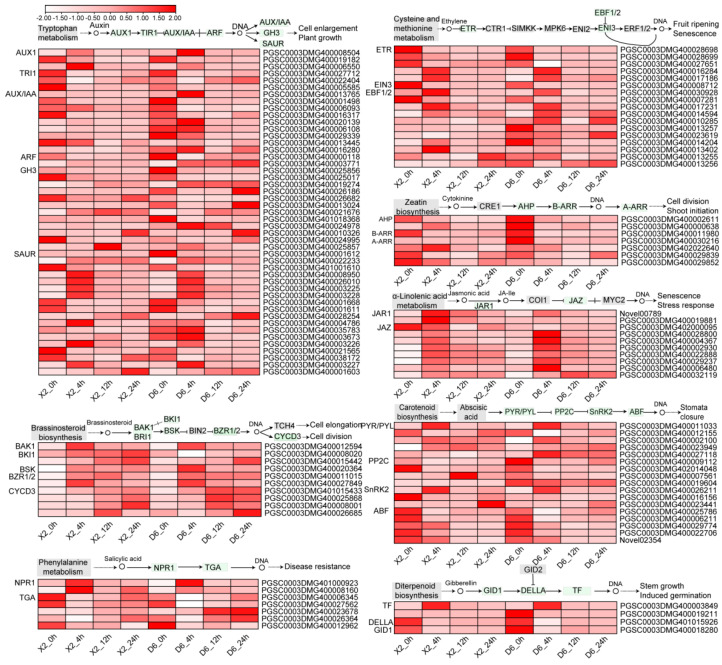
Heat map of DEGs involved in plant hormone signal transduction during enzymatic browning of potato tubers. Red represents a high transcript level and white represents a low transcript level. Dark gray areas represent missing values. BAK1: ETR: ethylene receptor; EBF1/2: EIN3-binding F-box protein; EIN3:ethylene-insensitive protein 3; ERF1/2: ethylene-responsive transcription factor 1; AUX1: auxin transporter-like protein; TIR1: TRANSPORT INHIBITOR RESPONSE 1-like; AUX/IAA: auxin/indole-3-acetic acid 3; GH3: indole-3-acetic acid-amido synthetase; SAUR: auxin-induced protein 15A-like; PYR/PYL: abscisic acid receptor PYL8-like; CPP2C: protein phosphatase 2; SnRK2: serine/threonine-protein kinase SAPK1-like; ABF: ABRE binding factor; AHP: histidine kinase 3; B-ARR: two-component response regulator ARR2-like; A-ARR: two-component response regulator ARR17-like; DID1: gibberellin receptor GID1; DELLA: DELLA protein RGL2-like; TF: transcription factor PIF4; BAK1: BRASSINOSTEROID INSENSITIVE 1-associated receptor kinase 1-like; BKI1: systemin receptor SR160; BSK: serine/threonine-protein kinase; BZR1/2: BES1/BZR1 homolog protein 2-like; CYCD3: cyclin D3; JAR1: jasmonic acid-amino synthetase; JAZ: jasmonate ZIM domain-containing protein; NPR1: regulatory protein NPR1; TGA: transcription factor TGA.

Eighteen ET-transduction-related genes were detected in the two cultivar tubers, including the genes encoding ethylene receptor (ETR), EIN3-binding F-box protein (EBF1/2), ethylene-insensitive protein 3 (EIN3), and ethylene-responsive transcription factor 1 (ERF1/2) (Figure 9). In general, the expression levels of these genes in both cultivars were higher at 0 h of cutting, followed by low expression levels at 12 h, and an increase thereafter. In both cultivars, among the ET-transduction-related genes, two genes (PGSC0003DMG400016284 and PGSC0003DMG400017186) encoding ETR, one gene (PGSC0003DMG400030928) encoding EBF1/2, and three genes (PGSC0003DMG400014594, PGSC0003DMG400013402, and PGSC0003DMG400017231) encoding ERF1/2 were significantly up-regulated after 4 h of cutting followed by a decrease, whereas the other genes were only down-regulated. The expression of two down-regulated genes encoding ETR and EIN3 (PGSC0003DMG400028698 and PGSC0003DMG400008712) significantly decreased after 4 h of cutting, especially the EIN3 gene which showed the highest expression in potato tubers (fragments per kilobase of transcript per million fragments mapped (FPKM) was 521.8 and 168.5 in the X2 and D6 tubers, respectively, but dramatically decreased in response to cut-wounding.

Forty-seven AUX-transduction-related DEGs were detected in both cultivar tubers, respectively, which included the genes encoding auxin transporter-like protein (AUX1), transport inhibitor response 1-like (TIR1), auxin/indole-3-acetic acid (AUX/IAA), indole-3-acetic acid-amido synthetase (GH3), and auxin-induced protein 15A-like (SAUR) belonging to the SAUR family of proteins. Similar to ET, the expression levels of most AUX-transduction-related genes were the highest at 0 h and the lowest at 12 h, with an increase thereafter. Of these genes, three AUX1 genes were identified. Among them, two genes were up-regulated after 4 h of cutting, followed by a decrease in both X2 and D6 tubers. Two of the TIR1 genes were down-regulated after cutting. Among the 10 AUX/IAA genes, the expression level of one gene (PGSC0003DMG400001498) significantly decreased during enzymatic browning after cutting with its log_2_FC being less than −4.27. Twelve GH3 gene transcripts were detected, of which eleven were differentially expressed in the D6 tubers, and eight in the X2 tubers. Twenty-two SAUR family genes were detected, of which, fifteen were differentially expressed in the D6 tubers and eighteen in the X2 tubers.

Seventeen ABA signal transduction-related DEGs were identified, encoding ABA receptor PYL8-like (PYR/PYL), protein phosphatase 2C (PP2C), serine/threonine-protein kinase SAPK1-like (SnRK2), and ABRE binding factor (ABF), respectively. The expression patterns of most genes were similar in both D6 and X2 tubers. However, the gene (PGSC0003DMG400027118) encoding PYR/PYL was up-regulated in the D6 tubers and down-regulated in the X2 tubers. PGSC0003DMG400007561 and PGSC0003DMG400019604 encoding PP2C were down-regulated in the D6 tubers and up-regulated in the X2 tubers.

Seven CK signal transduction-related genes were identified, encoding histidine kinase 3 (AHP), two-component response regulator ARR2-like (B-ARR), and two-component response regulator ARR17-like (A-ARR). Among them, PGSC0003DMG400000638 was down-regulated in the D6 tubers and up-regulated in the X2 tubers.

Four GA signal transduction-related genes were identified, encoding gibberellin receptor GID1 (DID1), DELLA protein RGL2-like (DELLA), and transcription factor (TF) PIF4. The gene PGSC0003DMG400003849 encoding GID1 was up-regulated at 4 h of cutting, followed by a decrease, while three genes encoding DELLA and TFs were significantly down-regulated.

Ten BR signal transduction-related genes encoding brassinosteroid-insensitive 1-associated receptor kinase 1-like (BAK1), systemin receptor SR160 (BKI1), serine/threonine-protein kinase (BSK), BES1/BZR1 homolog protein 2-like (BZR1/2), and cyclin D3 (CYCD3) were identified. Most genes were up-regulated in both cultivar tubers during enzymatic browning, except for PGSC0003DMG400008020 encoding BKI1, which was significantly down-regulated after 4 h of cutting in the D6 tubers.

Ten JA signal transduction-related genes encoding JA-amino synthetase (JAR1) and jasmonate ZIM domain-containing protein (JAZ) were identified. All genes were up-regulated except for PGSC0003DMG402000095 in both cultivar tubers during enzymatic browning.

Seven SA signal transduction-related genes encoding regulatory protein NPR1 (NPR1) and TF TGA were identified. Two or three of these genes were up-regulated, while three or four genes were down-regulated during enzymatic browning. In particular, PGSC0003DMG400023678 was continuously up-regulated at 0–24 h.

It was observed that almost all BR and JA signal transduction-related genes were up-regulated during enzymatic browning after cutting, whereas almost all the CK, GA, and SA signal transduction-related genes were down-regulated.

### 2.7. WGCNA Reveals Gene Modules Associated with Enzymatic Browning of Potato Tubers

The WGCNA showed that the modules of pink, tan, and black showed significant positive or negative correlation with PPO activity with relative coefficient of 0.8, 0.8 and −0.82, respectively; POD activity was positively correlated with a gray module with correlation coefficient of 0.84; CAT activity was negatively correlated with a green module, and the correlation coefficient was −0.95; PAL activity was positively and negatively correlated with blue and brown modules with the correlation coefficients were 0.92 and −0.83, respectively; a pink module is positively correlated with H_2_O_2_ (0.84) and negatively correlated with ΔL^*^. It is worth noting that a pink module is positively correlated with PPO, CAT, SOD, PAL activity and H_2_O_2_ content, but negatively correlated with ΔL^*^ (Figure S4), implying that the DEGs of the pink module were more important for enzymatic browning of potato tubers. Further, KEGG analysis was performed to reveal the function of DEGs of three modules significantly related to PPO, the result showed that the DEGs of the pink module are mainly concentrated in the synthesis and metabolism of fatty acids, and peroxisome; the DEGs of tan module are mainly concentrated in the metabolism of various amino acids; the DEGs of the black module were mainly enriched in the Ribosome and Phagosome pathways (Figure S5). Combined with the above results, it can be speculated that fatty acid metabolism and amino acid metabolism may play an important role in enzymatic browning of potato tubers. Furthermore, among the 172 DEGs in the pink module, 7 were transcription factor genes; in the blank module, 29 were transcription factor genes; and in the tan module, 2 were transcription factor genes. This implies that these transcription factor genes may be related to the expression regulation of genes associated with enzymatic browning (Table S11). In addition, we found a gene of interest in the pink module, a gene encoding Laccase (LAC, PGSC0003DMG401027116), which expression level increased rapidly after cutting and higher was in D6 tubers (FPKM is 2.71, 37.64, 83.69, 113.37, respectively) than X2 (FPKM is 2.91, 8.28, 45.34, 34.58, respectively), which is consistent with the result of qRT-PCR (Figure S6). In previous reports, the LAC plays an important role in the browning of apple and lychee peel [16]. Therefore, this gene may also play an important role in enzymatic browning of potato tubers.

### 2.8. Changes in Metabolites in Potato Tubers After Browning

To systemically reveal the metabolites alteration after browning, the metabolome profile of D6 tubers at 0 h and 24 h after cutting were performed using an untargeted metabolomics approach based on liquid chromatography-mass spectrometry (LC-MS). After removing the outlier, the result of PCA analysis showed that the samples from two time points were closely clustered together, respectively, indicating that the experiment was stable and repeatable. Hierarchical clustering for the expression levels of all metabolites intuitively demonstrated the stability of biological duplication (Figure S7).

**Figure 10:**
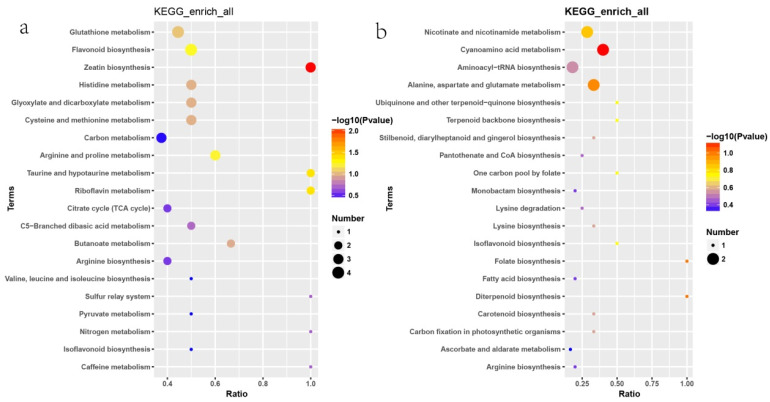
The KEGG enrichment analyses of DMAs. (**a**) Up-regulated DAMs; (**b**) down-regulated DAMs.

A total of 812 metabolites were detected. Among them, 197 Differentially Accumulated Metabolites (DAMs) were obtained (fold change ≥ 1.5 or ≤0.665, *p*-value < 0.05, VIP > 1), with 142 and 55 up- and down-regulated DAMs, respectively (Table S12). Among them, the most is lipids and lipid-like molecules with 28 metabolites; secondly is phenylpropanoids and polyketides and organic acids and derivatives, with 18 and 15 metabolites, respectively; the others include twelve organoheterocyclic compounds, eleven nucleosides, nucleotides, and analogs; ten organic oxygen compounds; ten benzenoids; two lignans, neolignans, and related compounds; one alkaloids and derivatives; one organic nitrogen compounds. The result of KEGG analysis showed that the up-regulated DAMs significantly enriched in the pathway of Zeatin biosynthesis. The pathway of taurine and hypotaurine metabolism, riboflavin metabolism, flavonoid biosynthesis, and arginine and proline metabolism were also significantly enriched (Figure 10a). The down-regulated DMAs were significantly enriched in the pathway of cyanoamino acid metabolism (Figure 10b).

Furthermore, we selected up-regulated metabolites with |log_2_FC| > 4 and down-regulated metabolites with |log_2_FC| > 2. The up-regulated DMAs selected including tetranor-PGFM (5.93), pterosin G (5.62), monobutyl phthalate (4.59), Timosaponin A1 (6.53), *p*-Coumaric acid ethyl ester (4.31), 3,4,5-Trimethoxycinnamic acid (4.31), N-Feruloyl putrescine (4.52), Selgin C-hexoside (4.35), chlortetracycline (4.32), 1,7-Diphenylhept-4-en-3-one (4.65), capsaicin (4.20), tyramine (4.49), naringenin (4.60); the down-regulated DMAs selected including 4-(acetylamino)phenyl 6-(trifluoromethyl)pyridine-2-carboxylate (−2.92), Coixol (−2.16). All of these metabolites may play important roles in enzymatic browning of potato tubers.

## 3. Discussion

Mechanical damage frequently occurs in fresh-cut fruits and vegetables during postharvest processing. On being damaged, fruits and vegetables undergo a series of physiological and sensory changes, among which is the enzymatic browning of tissues. In this study, RNA-seq was performed at different time points (early, middle, and late stages) in response to cut-wounding on two potato cultivars, namely a BR cultivar (X2) and a BS cultivar (D6), to understand the transcriptional response of the two potato tubers to cut-wounding and identify their differences in the possible mechanisms of enzymatic browning.

### 3.1. Response of DEGs to Cut-Wounding and the Differences Between BR and BS Cultivars

In this study, a total of 1248 DEGs, which represented overlapping DEGs at different time points after cutting in both X2 and D6 tubers, were identified. GO analysis showed that these DEGs were significantly enriched in the GO terms of redox-related functional terms (GO:0016491, GO:0016614, and GO:0016616), oxidation-reduction process (GO:0055114), and single-organism process (GO:0044699). KEGG analysis showed that these DEGs mainly participated in the pathways of DNA replication, stilbenoid, diarylheptanoid, and gingerol biosynthesis, flavonoid biosynthesis, mismatch repair, biosynthesis of secondary metabolites, biotin metabolism, and phenylalanine metabolism. The results of the expression trend analysis showed that the DEGs of the pathways of stilbenoid, diarylheptanoid, and gingerol biosynthesis, flavonoid biosynthesis, phenylalanine metabolism, phenylpropanoid biosynthesis, biotin metabolism, and biosynthesis of secondary metabolites, all of which are the main source of phenolic compounds, were up-regulated in response to cut-wounding. Down-regulated DEGs clustered in profile 2 mainly participated in the pathways of the spliceosome, mismatch repair, and DNA replication. These overlapping DEGs were functionally categorized into a variety of physiological and molecular processes, which revealed the conserved mechanisms of wounding-responsive genes in potato tubers. Genotype-specific DEGs of the X2 and D6 tubers revealed the differences in the enzymatic browning process between BS and BR tubers. The results of our study showed that the BS cultivar D6 tubers had faster redox-related reactions. Smith et al. (2004) showed that the up-regulated genes were related to plant defense mechanisms, while the down-regulated ones were related to programmed cell death after plant tissue damage [17].

### 3.2. The Cut-Wounding Promotes the Biosynthesis of Phenolics, Flavonoids, and Lipids

Both DEGs and DAMs were significantly enriched in lipid metabolism and biosynthesis, phenolic biosynthesis-related pathways, and flavonoid biosynthesis in potato tubers after cutting. Therefore, these pathways may play an important role in the browning process of potato tubers, which is consistent with the results of Qiao et al. (2022) and Jiang et al. (2022) [18,19]. Phenylpropanoid biosynthesis is the principal pathway for phenol and lignin biosynthesis. It is also the substrate source for flavonoid synthesis and other metabolic pathways [20]. Biosynthesis of phenols is an important feature of plant wounding response. One of the most detrimental changes induced by wounding is the induction of phenylpropanoid metabolism, which results in the accumulation of phenolic compounds and subsequent tissue browning [21]. PAL and C4H were induced and up-regulated by wounding in poplars [17]. Our previous study also showed that wounding increased the total phenolic content in potato tubers [15]. PAL is crucial for the initiation of the first committed step in the phenylpropanoid pathway and for the formation of trans-cinnamic acid which is involved in various wound response mechanisms. Being a key enzyme, the regulation of PAL activity is important to modulate phenylpropanoid biosynthesis in plants [10]. PAL genes expression was regulated by a diverse array of factors, including injury, infection, environment, and the developmental stage of plants [22,23,24,25]. It can catalyze the non-oxidative deamination of L-phenylalanine to form trans-cinnamic acid and a free ammonium ion. This reaction is the first step in the biosynthesis of a large range of phenylpropanoid-derived secondary products in plants, including flavonoids and isoflavonoids, coumarins, lignins, wound-protective hydroxycinnamic acid esters, and other phenolic compounds [10]. Wounding was reported to induce the PAL activity six- to twelve-fold over 24 h, leading to a three-fold increase in the total phenolic content within three days in iceberg lettuce leaves [21]. In this study, six PAL genes were identified and up-regulated by cut-wounding in both cultivars. The activity of PAL was increased rapidly after cutting. In the DAMs after browning, seven flavonoids were detected among the differential metabolites. Except epigallocatechin, the others, including (+)-catechin, (−)-epigallocatechin, hesperetin, naringenin, rhodionin, trifolin, all significantly increased. Three phenols detected were all increased, including 4-Hydroxymandelonitrile, capsaicin, N-Feruloyltyramine. Wang et al. (2023) [16] revealed that preharvest apple peel browning was primarily due to changes in phenolics and flavonoids.

### 3.3. Higher PPO, POD Activity and H_2_O_2_ Content Were the Main Reasons Why D6 Was More Prone to Browning than X2

A powerful antioxidant system was an important element in delaying browning [26]. Release of reactive oxygen species (ROS) is termed ‘oxidative burst’. It is a rapid, transient production of huge amounts of ROS, which is one of the earliest observable aspects of a plant’s defense strategy [27]. H_2_O_2_ is a relatively stable ROS and considered an important signaling molecule for plant development and response. It can pass through cell membranes and reach remote cell locations from the site of its formation. High levels of H_2_O_2_ can be observed in wounded cells or cells subjected to mechanical stress [28]. Excessive H_2_O_2_ leads to plant toxicity and hence, plants have evolved mechanisms to keep their levels balanced. POD, CAT, and SOD are important ROS-scavenging enzymes in plants. The POD gene is the last key gene involved in lignin synthesis and also one of the key enzymes for enzymatic browning in plant tissues. It is a thermostable enzyme that belongs to a group of oxidases that use H_2_O_2_ as a catalyst for the oxidation of phenolic compounds. In our study, 30 POD genes were identified, and more than 70% of them were up-regulated after cutting, and its enzyme activity declined after cutting. Four CAT genes were identified. Unlike other enzymatic browning-related genes, the expression levels of the CAT genes in the D6 tubers were lower compared to those in the X2 tubers, especially at 0 h after cutting. This showed that the BR cultivar X2 tubers may have a stronger ability to scavenge H_2_O_2_ compared to the BS cultivar D6 tubers and reduced the degree of enzymatic browning induced by POD. Interestingly, a recent study showed that the degree of enzymatic browning in potato tubers can be significantly reduced by treating them with a certain concentration of CAT, which reduced H_2_O_2_ and O^2−^ content. At the same time, CAT treatment was found to inhibit the activities of PPO, POD, and PAL and reduce phenol accumulation [29]. However, in our results, although X2 had higher expression levels of CAT genes than D6, its enzyme activity was lower than D6. Unlike with the CAT genes, most SOD gene expression levels in the X2 tubers were higher compared to those in the D6 tubers, which consistent with its higher enzyme activity. It can be seen that not all anti-browning materials have a powerful antioxidant system. PPO is the main enzyme responsible for enzymatic browning in plants. Unlike POD, which catalyzes the oxidation of phenolic compounds only in the presence of H_2_O_2_, PPO can directly catalyze monophenol or bisphenol to quinones, resulting in the browning of plant tissues. Recent studies show that there are two potential PPO substrates, namely flavonoids and phenolic acids [30]. Studies also show that POD can enhance the degree of browning of damaged tissues in the presence of PPO due to the generation of H_2_O_2_ by the oxidation of phenolic compounds [10]. In this study, consistent with the expression levels of PPO genes, its enzyme activity was also significantly higher in D6 tubers than X2 during enzymatic browning.

In short, we concluded that the BS cultivar D6 tubers were more prone to browning compared to the BR cultivar X2 tubers due to the presence of higher PPO and POD gene expression levels in the D6 tubers compared to the X2 tubers; the higher H_2_O_2_ content further promoted the POD catalytic phenolic compounds to quinone. Our previous results showed that compared with total phenol content, PPO and POD activities had greater effects on enzymatic browning of potato tubers [31].

### 3.4. LAC May Play an Important Role in Enzymatic Browning of Potato Tubers

Based on WGCNA, we found a LAC gene, another polyphenol oxidase gene, in the pink module, which was significant correlation with the PPO activity and ΔL^*^. The expression of LAC gene increased rapidly after potato tuber cutting, and higher in D6 than X2. Recent studies have shown the role of LAC in the browning of litchi, apple, and various other fruits during storage [32,33,34]. LACs, similar to PPO, can oxidize various compounds, including o-diphenols, *p*-diphenols, methoxy-substituted monophenols, diamines, and nonaromatics [34]. Compared with the PPO, LACs have a broader substrate range [35]. The Arabidopsis thaliana transparent testa10/12/16 (TT10/12/16, laccase-like enzyme) mutant exhibited a delay in development-related browning of the seed coat [36]. Wang et al. (2023) revealed the MdLAC7-mediated mechanism regulating preharvest apple peel browning. At present, there are no studies on the correlation between LAC and enzymatic browning in potato, which is worthy of further study [16].

### 3.5. Genes Related to Plant Hormone Signal Transduction May Be Involved in the Enzymatic Browning of Potato Tubers

Plants integrate multiple hormone-response pathways to adapt to biological and abiotic stress. They transport the wounding signal to adjacent tissues and induce changes in the related genes. When plants are injured, wounding signals are transmitted to neighboring tissues by phytohormones, including ET, ABA, SA, etc. [37]. Plant hormones, namely ABA and JA, are key components of the wound signal transduction pathway [38]. In the present study, eight kinds of hormone transduction-related genes were identified. Among them, 52 genes were found related to IAA signal transduction, which was far more than other hormone signal transduction-related genes. The number of ET and ABA signal transduction-related genes was 18 and 17, respectively. Other hormone transduction-related genes were less than or equal to ten genes. These results indicated that the IAA, ET, and ABA could play an important role in response to the wounding of potato tubers. Numerous studies have shown that ET and IAA play important roles in response to various biological and abiotic stresses, such as drought and injury. Researchers showed that ET participates in various stress responses and induces PAL biosynthesis [39]. Biosynthesis of ET is related to the aging of an organism, with aging tissues being prone to browning, e.g., bananas, dates et al. [10]. ET promotes the formation of wax, which in turn promotes wound healing, while high concentrations of IAA promote ET biosynthesis [40,41]. In addition, a previous study also showed that IAA plays an important role in the metabolic response to wounding [42]. Studies also show that ABA plays an important role in plant wound healing. ABA was found to regulate the healing ability of potato tubers after injury by stimulating the formation of the cork layer [43,44,45,46]. The injury induced the biosynthesis of ABA, which was necessary for the synthesis of lignin and phenol after injury [37,43]. The wounding of potato tubers increased ABA biosynthesis and catabolism and altered the expression of ABA metabolic genes [37]. ABA was also found to promote the formation of postharvest callus of sweet potato roots by significantly increasing the activities of PAL and the content of flavonoids and phenols [47]. ABA was studied to control the internal browning of pineapple by inhibiting phenol biosynthesis and oxidation and enhancing its antioxidant capability [48]. SA and JA are key signal molecules that regulate plant resistance [49]. Mechanical damage provokes de novo synthesis of PAL in plant tissues, probably through signaling pathways involving SA and JA [10]. Systemic wound response also contributes to the rapid synthesis of jasmonates to enhance plant resistance to herbivore and insect attacks [50]. Zhou et al. (2019) showed that methyl jasmonate promotes wound healing and enzymatic browning in fresh-cut potato cubes [51]. JA treatment was found to cause a significant increase in the activities of plasma membrane NADPH oxidase and PAL in pea leaves [52]. Compared to other hormones, the relationship between GA and enzymatic browning is rarely studied. However, in recent years, studies on the effects of GA on fruit and vegetable quality were made. For example, GA treatment was found to alleviate the browning of pericarp litchi during storage [53,54]. Aly and Ismail (2000) reported that the pre-harvest treatment of ‘Balady’ guava with GA3 (150 ppm) decreased fruit skin browning and polyphenol oxidase activity compared to the control and boron-treated fruits [55]. In this study, a total of four genes related to GA response were identified during the enzymatic browning of potato tubers. Except for one gene, the expression levels of the remaining genes decreased after cutting. Brassinosteroid was investigated for its possible role in the postharvest physiology of various horticultural crops. Studies showed that exogenous brassinosteroid can inhibit PPO activity, enhance POD, CAT, and ascorbate peroxidase (APX) enzyme activities, and reduce H_2_O_2_ accumulation, thereby decreasing the surface browning of fruits and vegetables [56,57,58]. It was also reported that brassinosteroid can accelerate wound healing in potato tubers by the activation of reactive oxygen metabolism and phenylpropanoid metabolism [59]. In this study, brassinosteroid signal transduction-related genes were found up-regulated.

## 4. Materials and Methods

### 4.1. Plant Materials

Two tetraploid potato cultivars Xingjia 2 (X2, a BR cultivar) and Dianshu 6 (D6, a BS cultivar) were used in this study. Both cultivars were grown in the experimental field of Baiyun District, Guangdong Province, China. Within 30 d after maturity and harvest, healthy tubers without visible disease or mechanical damage were selected, thoroughly washed under running water, dried, cut transversely into halves, and then keep in trays at 25 °C. At designated time points (0, 4, 12, and 24 h) after cutting, approximately 5 mm thick tissue blocks (radically sliced from epidermis to pith; Figure S8), flash freeze in liquid nitrogen, and stored at −80 °C for later use. Samples taken immediately after cutting (0 h) served as the control. Three biological replicates (three tubers per replicate) per cultivar were collected, and tuber tissue surface color (L*, a*, and b*) were measured using a colorimeter (NS810, 3nh, Shenzhen, China) calibrated with a standard white plate (L*_0_, a*_0_, b*_0_). The calculation of ΔL*, Δa*, and Δb* as follows: ΔL* = L*−L*_0_, Δa* = a*−a*_0_, Δb* = b*−b*_0_.

### 4.2. Determination of Enzyme Activity, Total Phenol and H_2_O_2_ Content

PPO, POD, PAL, CAT, SOD activities and H_2_O_2_ content were assayed calorimetrically using commercial assay kit (Product code: BC0190, BC0090, BC0210, BC4780, BC0170, BC3590, respectively; Beijing Solarbio Science & Technology Co. Ltd., Beijing, China) following the manufacturer’s instructions. For the determination of total phenol content, refer to Wang et al. (2020) [15].

### 4.3. RNA Extraction, RNA Sequencing (RNA-Seq), and Functional Annotations

The total RNA of the tissues was extracted TiangenRNA extraction kit (Tiangen Biltech Co. Ltd., Beijing, China) according to the manufacturer’s protocol, and integrity was verified by RNase-free agarose gel electrophoresis. The mRNA library was constructed and sequenced on the Illumina Hiseq platform using the paired-end reads method. Clean reads were generated by filtering raw data to remove adapter sequences, ploy-N reads, and low-quality reads. Quality metrics including Q20, Q30, and GC content were calculated for the clean data. The reads were then aligned to the potato reference genome (PGSC_DM_v3.4) [60] using HISAT [61]. Gene-level read counts were obtained using FeatureCounts (v1.5.0-p3). Subsequently, FPKM values for each gene were calculated based on gene length and mapped reads counts. To gain insights into the changes in phenotype, enrichment analysis of the DEGs, the GO enrichment analysis was performed using the TopGO R package and AgriGO program [62,63], and the KEGG (https://www.kegg.jp/ (accessed on 20 December, 2024)) pathway was conducted using KO-BAS3.0 with an enrichment *p*-value < 0.05.

### 4.4. Quantitative Real-Time PCR (qRT-PCR) Analysis

First-strand cDNA was synthesized from 2 μg total RNA using Tiangen TransScript reverse transcriptase. qRT-PCR was conducted using Premix Ex TaqTM (Takara, Japan) on an Applied Biosystems StepOnePlus thermocycler (Applied Biosystems, Beverly, MA, USA). EF1α was used as the internal control [64]. The 2^−ΔΔCt^ method was used to analyze the relative changes in gene expression. The expression assay was repeated three times, and each assay was performed with three independent technical replicates. The information of the primers is listed in Table S13.

### 4.5. Weighted Gene Co-Expression Network Analysis (WGCNA)

WGCNA (R pakage, https://github.com/ShawnWx2019/WGCNA-shinyApp (accessed on 2 November 2024)) was performed to construct co-expression networks of all DEGs. The soft thresholding power was chosen based on the lowest power for which the scale-free topology fit index reached a high value. We estimated the Pearson correlation coefficients among the genes based on their FPKM values by converting the correlation matrix into an adjacency matrix. Hierarchical clustering and dynamic tree cut function were used to detect modules, grouping all genes into clusters. Gene significance (GS) and module membership (MM) were calculated to correlate the modules with the phenotypic data. The information of the corresponding module genes was extracted for further analysis.

### 4.6. Metabolome Profiling

The surface tissue of potato tubers at 0 h and 24 h after cutting was selected for metabolome profiling, and six biological replicates were prepared for each time point. Metabolites extraction, separation, identification, and data treating were performed using a Waters 2D Ultra Performance Liquid Chromatography (Thermo Fisher Scientifc, Cleveland, OH, USA) equipped with a Q Exactive high-resolution mass spectrometer (Thermo Fisher Scientifc, Cleveland, OH, USA). MS data were obtained in electrospray ionization-negative (ESI−) and electrospray ionization-positive (ESI+) modes to improve metabolite coverage. LC-MS/MS data were processed using Compound Discoverer 3.3 (Thermo Fisher Scientifc, Cleveland, OH, USA) software, mainly including peak extraction, peak alignment, and compound identification. The differences in compounds were identified using Metabo Analyst 4.0 software. The metabolites with fold change ≥ 1.5 or fold change ≤ 0.667 and *p* < 0.05 were identified as DAMs. KEGG database was used for significant enrichment analyses and functional classification of DAMs.

### 4.7. Statistical Analysis

The statistical analysis was performed using Microsoft Excel 2010 and IBM SPSS 25.0. Charts were drawn by OriginPro 9.1 software. The means among various groups were compared by Duncan’s multiple range tests. The data were analyzed and are expressed as the means ± standard deviations (SDs), and *p* < 0.05 indicated significance differences.

## 5. Conclusions

In this study, samples of a BS and BR potato cultivar tubers were collected at different time points (0 h, 4 h, 12 h, and 24 h) during enzymatic browning after cutting. The responses of the two different browning phenotypes of potato tubers to cut-wounding were, in general, similar. The pathways of DNA replication, stilbenoid, diarylheptanoid, and gingerol biosynthesis, flavonoid biosynthesis, mismatch repair, biosynthesis of secondary metabolites, biotin metabolism, and phenylalanine metabolism show significant response to cut-wounding in both X2 and D6 tubers. The up-regulated DEGs mainly related to the secondary metabolite synthesis, and the down-regulated DEGs related to the programmed cell death. Function analysis of specific DEGs at different time points after cutting showed that the BS tubers had earlier significant redox-related reactions compared to the BR tubers. Phenolic, lipid, and flavonoid compounds accumulated during the browning of potato tubers. The higher PPO, POD activity, and H_2_O_2_ contents were the main reasons that D6 more easily browned, and the BR cultivar X2 did not show significantly stronger antioxidant capacity than BS cultivar D6. In addition, a LAC gene, which has been related to browning in previous studies, was found in potato tubers after cutting and was worthy of further study. Plant hormones may also be a focus of attention in the future.

## Figures and Tables

**Table 1:** GO annotation of 1248 overlapping differentially expressed genes of potato tubers at different times after cutting.

GO_Accession	Description	Term_Type	DEG Number	qValue
GO:0016491	oxidoreductase activity	MF	181	8.04 × 10^−5^
GO:0016614	oxidoreductase activity, acting on CH-OH group of donors	MF	35	0.015521
GO:0016616	oxidoreductase activity, acting on the CH-OH group of donors, NAD or NADP as acceptor	MF	32	0.019845
GO:0015238	drug transmembrane transporter activity	MF	14	0.041072
GO:0090484	drug transporter activity	MF	14	0.041072
GO:0055114	oxidation-reduction process	BP	172	0.000327
GO:0042493	response to drug	BP	14	0.041072
GO:0044699	single-organism process	BP	494	0.041072

**Table 2:** GO enrichment of specific DEGs of X2 and D6 tubers at different times after cutting.

The Type of DEGs	Samples	GO Enrichment
**4 h after cut-wounding**		
Up regulation	X2_specific	protein phosphorylation, cellular protein, modification process, protein modification process, transferase activity, transferring hexosyl groups, transferase activity, transferring glycosyl groups, transferase activity
	D6_specific	oxidation-reduction process, single-organism metabolic process, oxidoreductase activity
Down regulation	X2_specific	ADP binding, transition metal ion binding, cation binding, metal ion binding
	D6_specific	ADP binding, transition metal ion binding, cation binding, metal ion binding
**12 h after cut-wounding**		
Up regulation	X2_specific	catalytic activity, transferase activity, oxidoreductase activity
	D6_specific	metabolic process, organic substance metabolic process, cellular metabolic process, metabolic process, organic substance metabolic process, cellular metabolic process, nitrogen compound metabolic process, cellular nitrogen compound metabolic process, gene expression, cytoplasmic part, intracellular organelle, organelle cytoplasm
Down regulation	X2_specific	intracellular organelle part, organelle part, monovalent inorganic cation transport, monovalent inorganic cation transmembrane transporter activity
	D6_specific	cation binding, metal ion binding, ion binding, regulation of RNA biosynthetic process, regulation of RNA metabolic process, transcription, DNA-templated, regulation of transcription, DNA-templated, regulation of nucleic acid-templated transcription
**24 h after cut-wounding**		
Up regulation	X2_specific	catalytic activity, single-organism metabolic process, oxidation-reduction process, oxidoreductase activity, transferase activity, metabolic process
	D6_specific	oxidoreductase activity, oxidation-reduction process, catalytic activity, metabolic process, single-organism metabolic process, single-organism process
Down regulation	X2_specific	cation binding, metal ion binding, nucleic acid binding
	D6_specific	ion binding, regulated-related

## Data Availability

The RNA-seq data of the present investigation were submitted to the SRA database under the bioproject ID SRP324904.

## Supplementary Materials

The following supporting information can be downloaded at: https://www.mdpi.com/article/10.3390/plants14121817/s1, Figure S1: Correlation matrix of samples; Figure S2: Validation of qRT-PCR to RNA-seq; Figure S3: GO enrichment analysis of DEGs of samples at different time; Figure S4: Module−trait relationships; Figure S5: Figure S5 KEGG analysis of DEGs of pink, tan, black module; Figure S6: The expression level of laccase gene; Figure S7: Figure S7 Principal component analysis of samples and analysis; Figure S8: Schematic diagram of RNA-seq sampling of potato tubers; Table S1: Summary of transcriptome sequencing data; Table S2: mapping rate of RNA-Seq data; Table S3: gene information of qRT-PCR; Table S4: Distribution of DEGs in different functional groups based on GO enrichment; Table S5: KEGG enrichment analysis of 1248 common DEGs; Table S6: The KEGG enrichment analysis of DEGs of Profile17 and Profile 2; Table S7: GO enrichment analysis of specific DEGs at different time after cutting; Table S8: Key gene expression of the pathway of Phenylpropanoid biosynthesis based on RNA-seq; Table S9: Antioxidant enzyme-related genes expression based on RNA-seq; Table S10: The expression of DEG-related plant hormone signal transduction based on RNA-seq. Table S11: The genes information of different models; Table S12: Differentially Accumulated Metabolites; Table S13: Primer information of qRT-PCR.
