# Supplementary material for: Comparative Transcriptome and Metabolome Profiling Revealed Molecular Cascade Events During the Enzymatic Browning of Potato Tubers After Cutting
Source: Plants (Basel). 2025 Jun 13;14(12):1817. doi: 10.3390/plants14121817 (PMC12196699; doi:10.3390/plants14121817)
Supplement: Supplementary file 1 [file plants-14-01817-s001.zip › Figure S3 GO enrichment analysis of DEGs of samples at different time.pdf]

D6_4 h vs D6_0 h

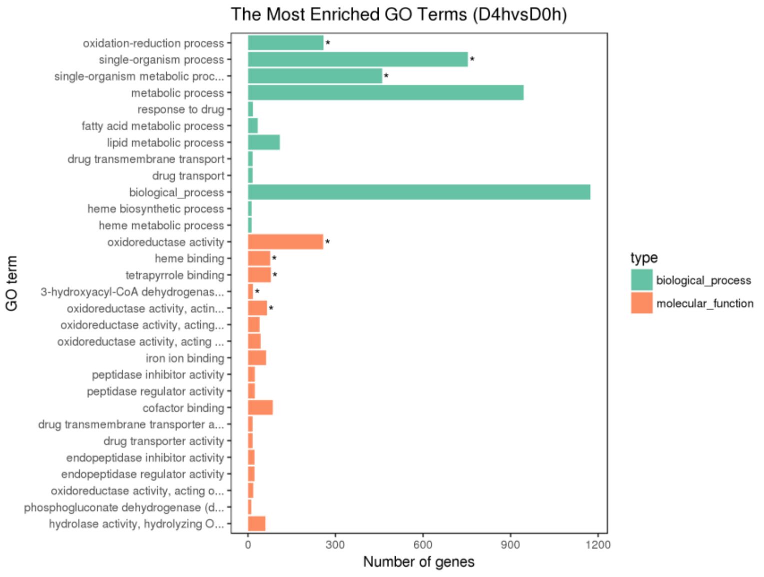

D6_12 h vs D6_0 h

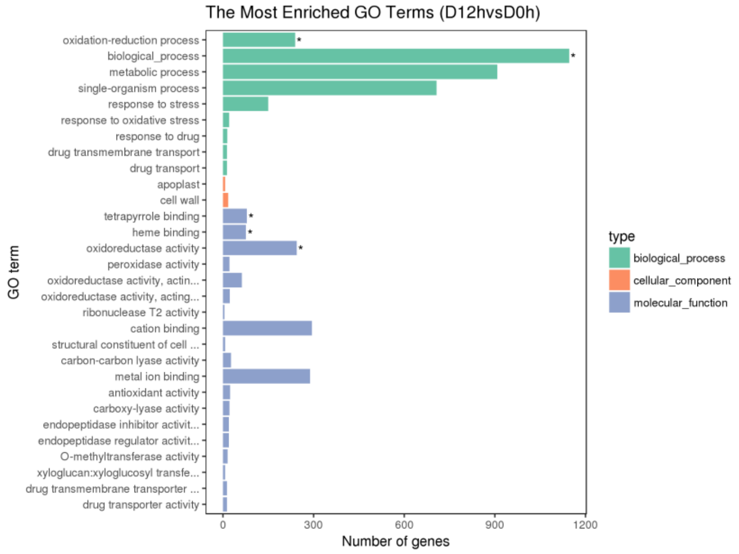

D6_24 h vs D6_0 h

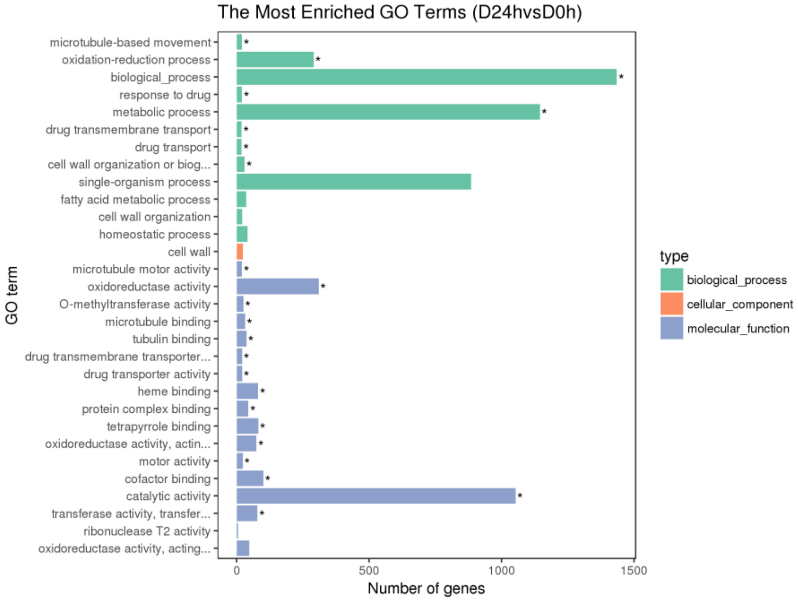

X2_4 h vs X2_0 h

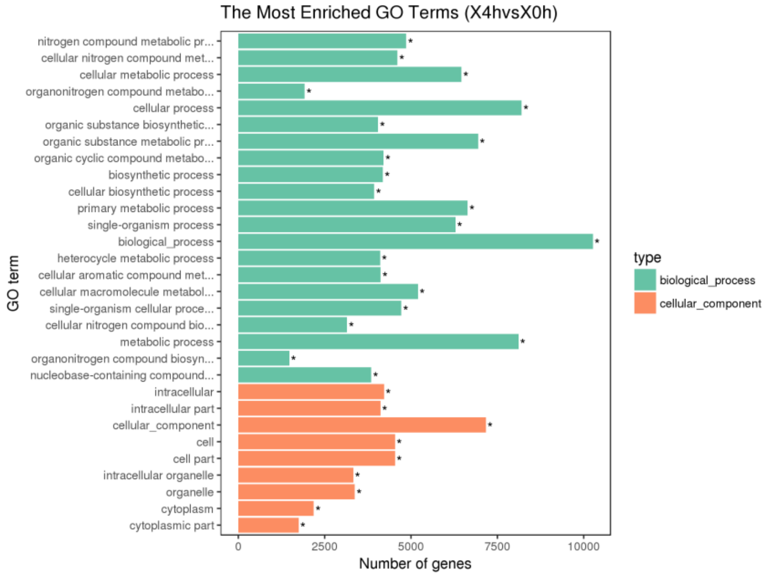

X2_12 h vs X2_0 h

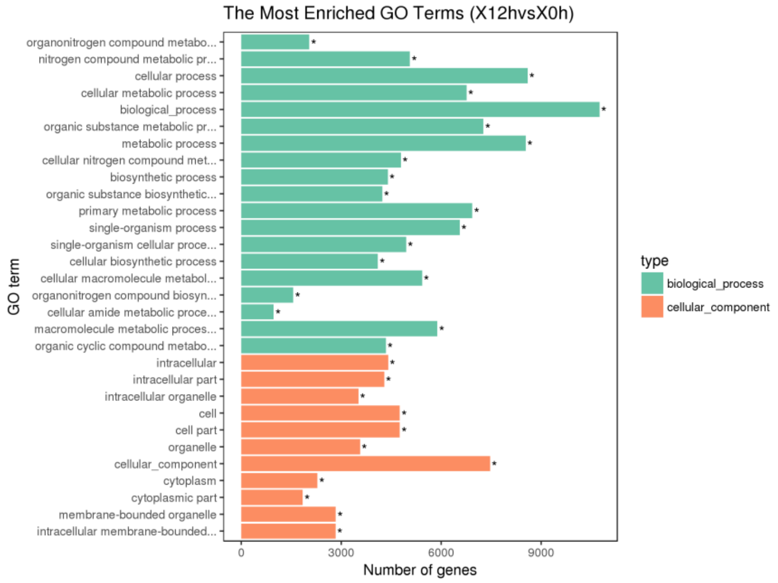

X2_24 h vs X2_0 h

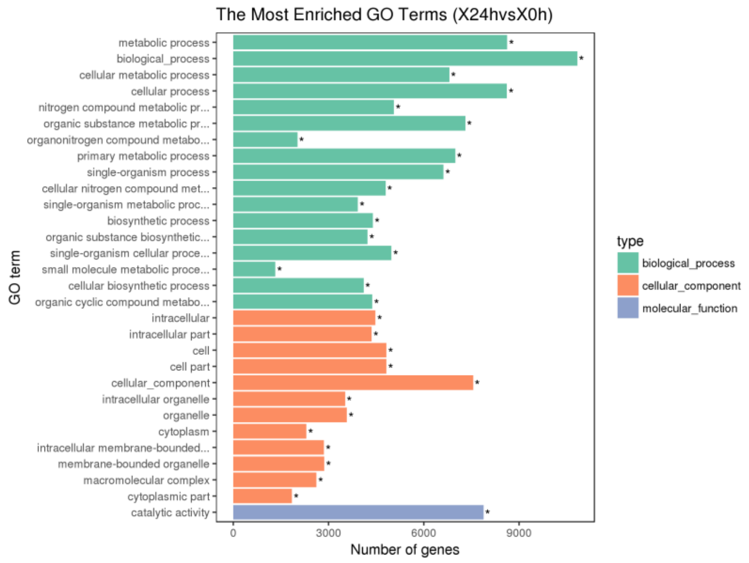

Figure S3. GO enrichment analysis of DEGs of samples at different time
