# Supplementary material for: Comparative Transcriptome and Metabolome Profiling Revealed Molecular Cascade Events During the Enzymatic Browning of Potato Tubers After Cutting
Source: Plants (Basel). 2025 Jun 13;14(12):1817. doi: 10.3390/plants14121817 (PMC12196699; doi:10.3390/plants14121817)
Supplement: Supplementary file 1 [file plants-14-01817-s001.zip › Figure S7 Principal component analysis of samples and analysis.pdf]

a.

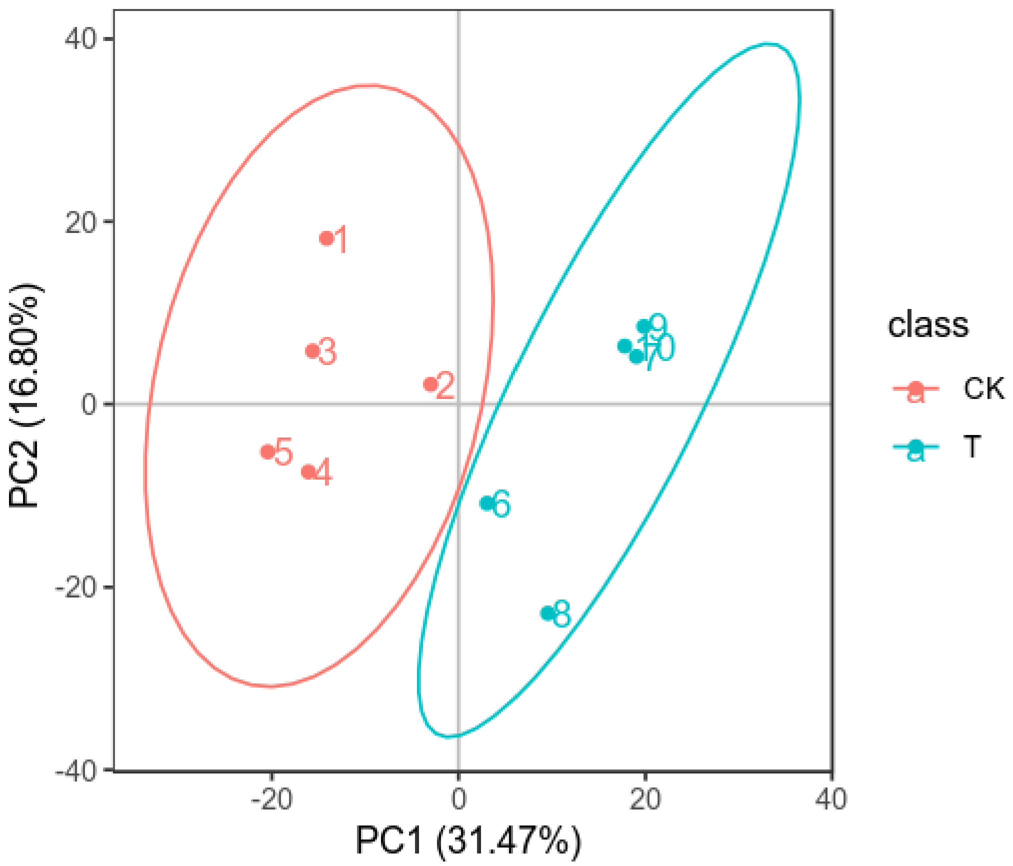

b.

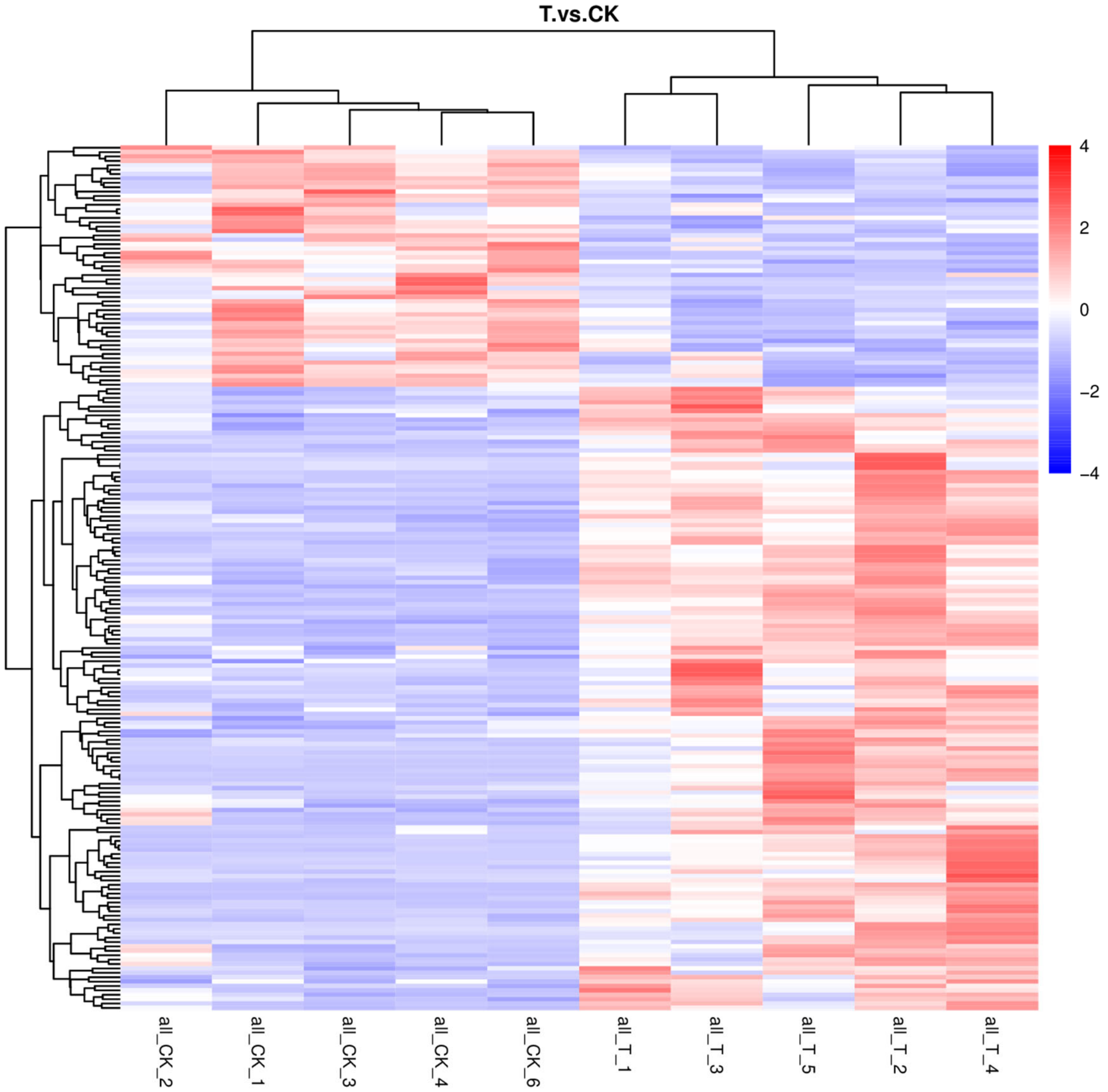


Figure S7 Principal component analysis of QC samples and analysis of metabolite strength distribution. Note: a: PCA model figure; b: Hierarchical clustering
