# Supplementary figures and images for: Comparative Transcriptome and Metabolome Profiling Revealed Molecular Cascade Events During the Enzymatic Browning of Potato Tubers After Cutting
Source: Plants (Basel). 2025 Jun 13;14(12):1817. doi: 10.3390/plants14121817 (PMC12196699; doi:10.3390/plants14121817)

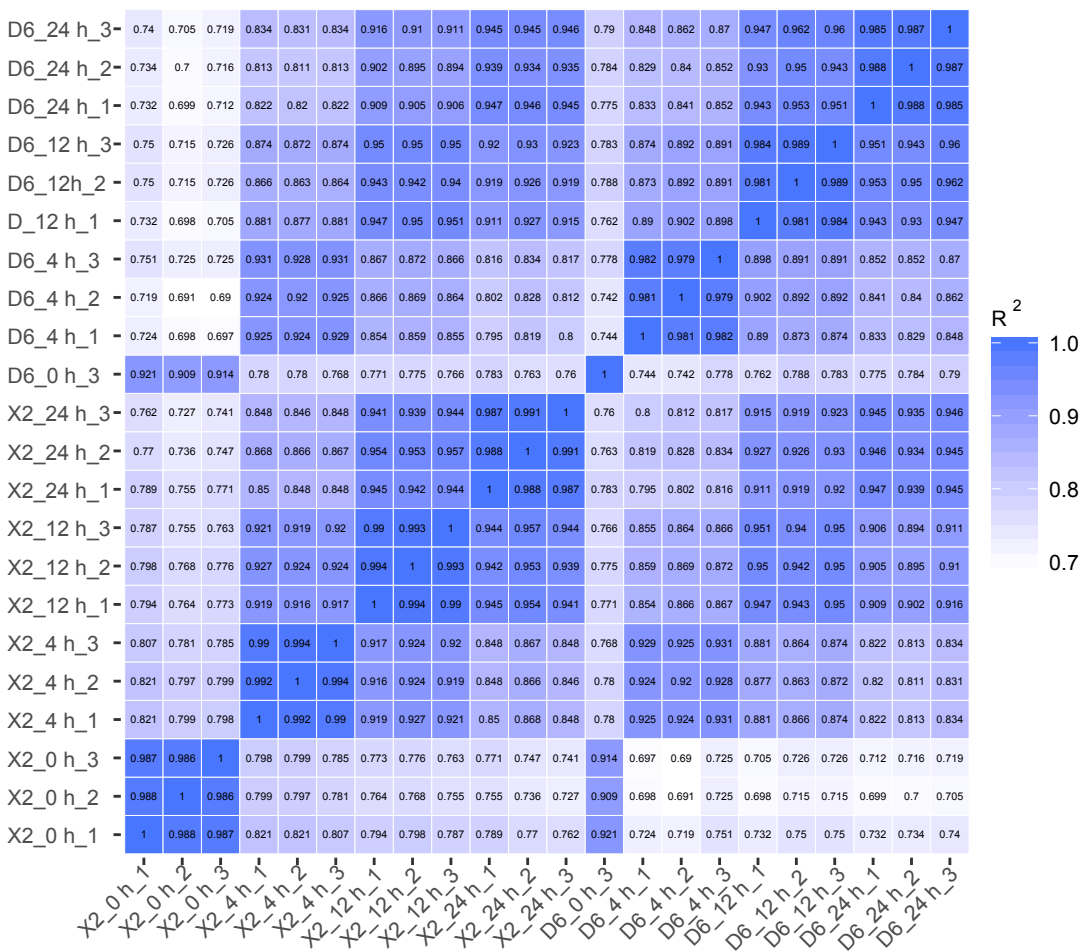

Supplement: Supplementary file 1 [file plants-14-01817-s001.zip › Figure S1 Correlation matrix of samples.pdf]

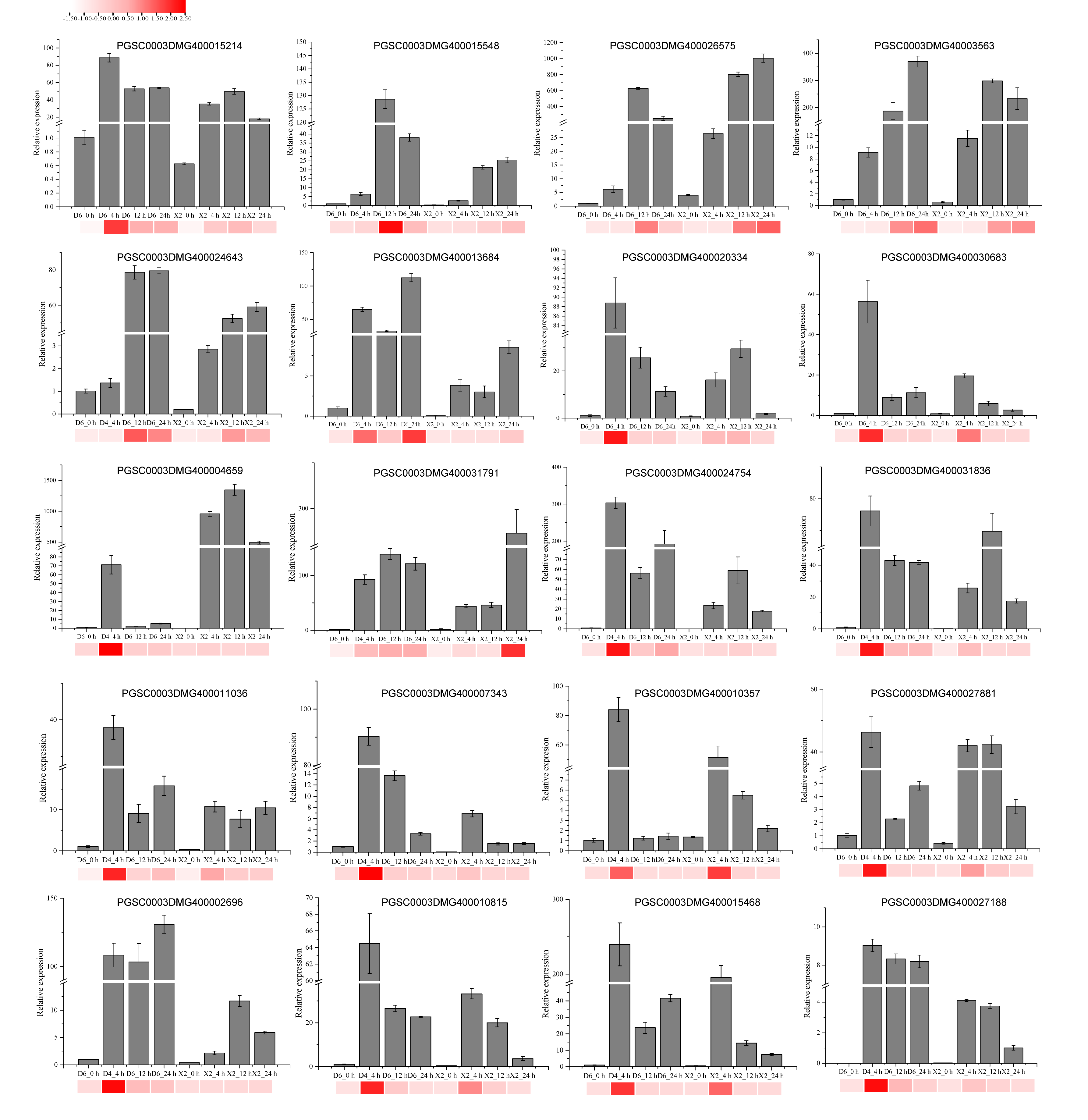

Supplement: Supplementary file 1 [file plants-14-01817-s001.zip › Figure S2 Validation of qRT-PCR to RNA-seq.tif]

Module–trait relationships

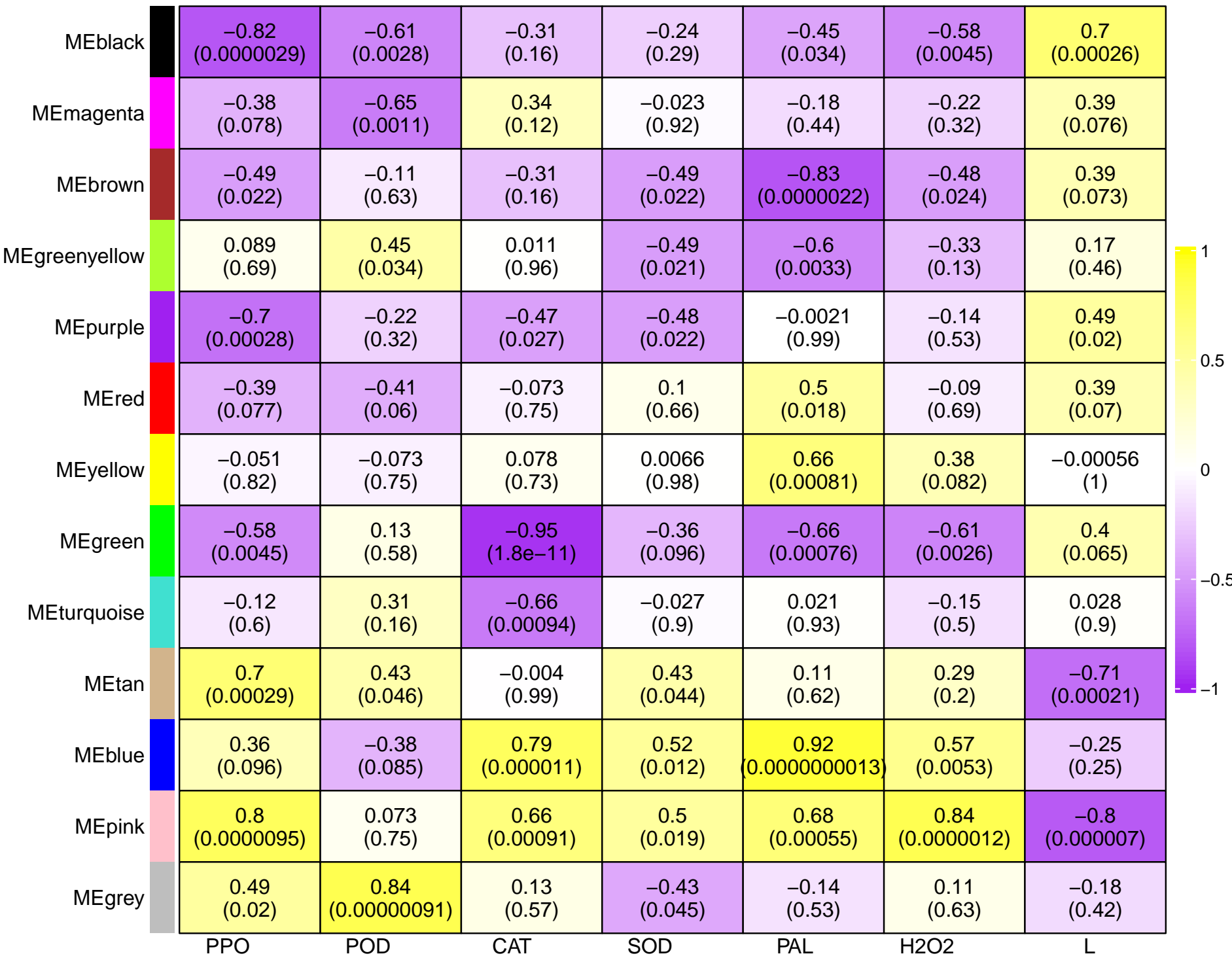

Supplement: Supplementary file 1 [file plants-14-01817-s001.zip › Figure S4 Module-trait relationships.pdf]

Pink module

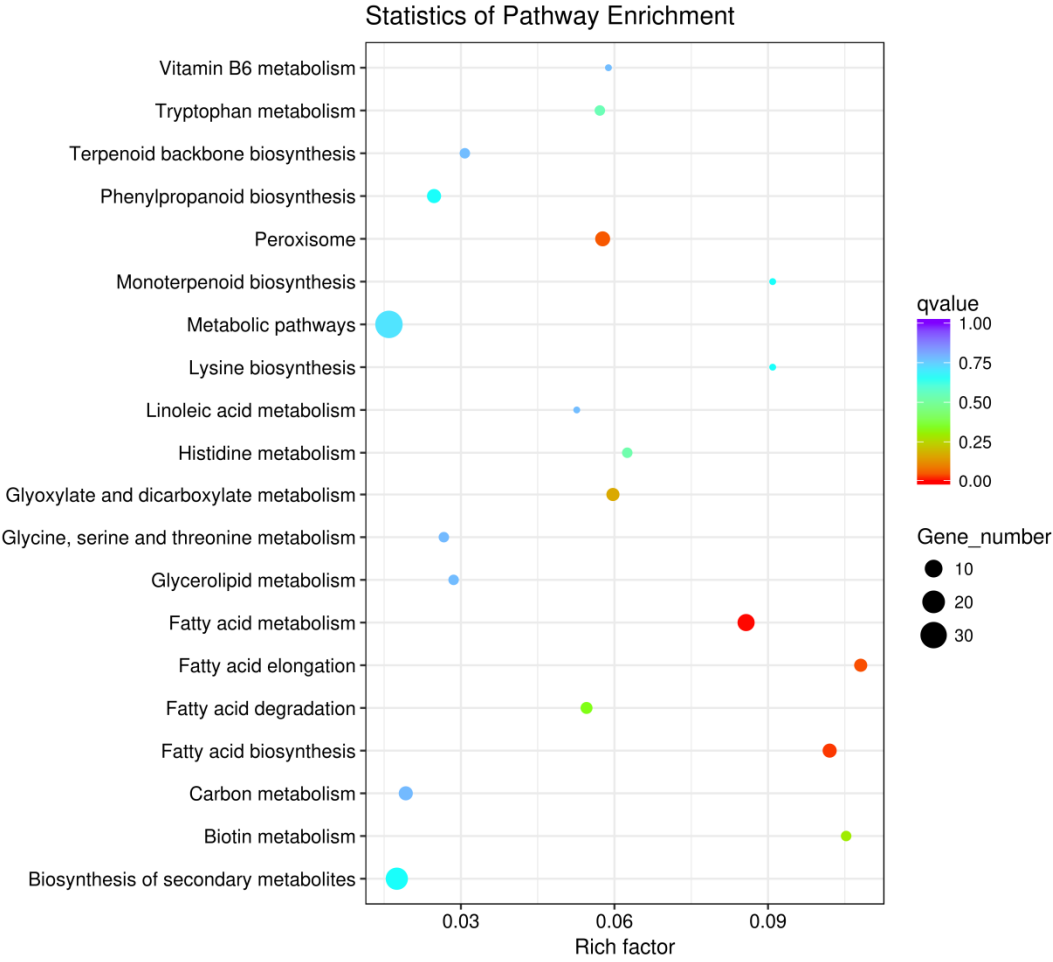

Tan module

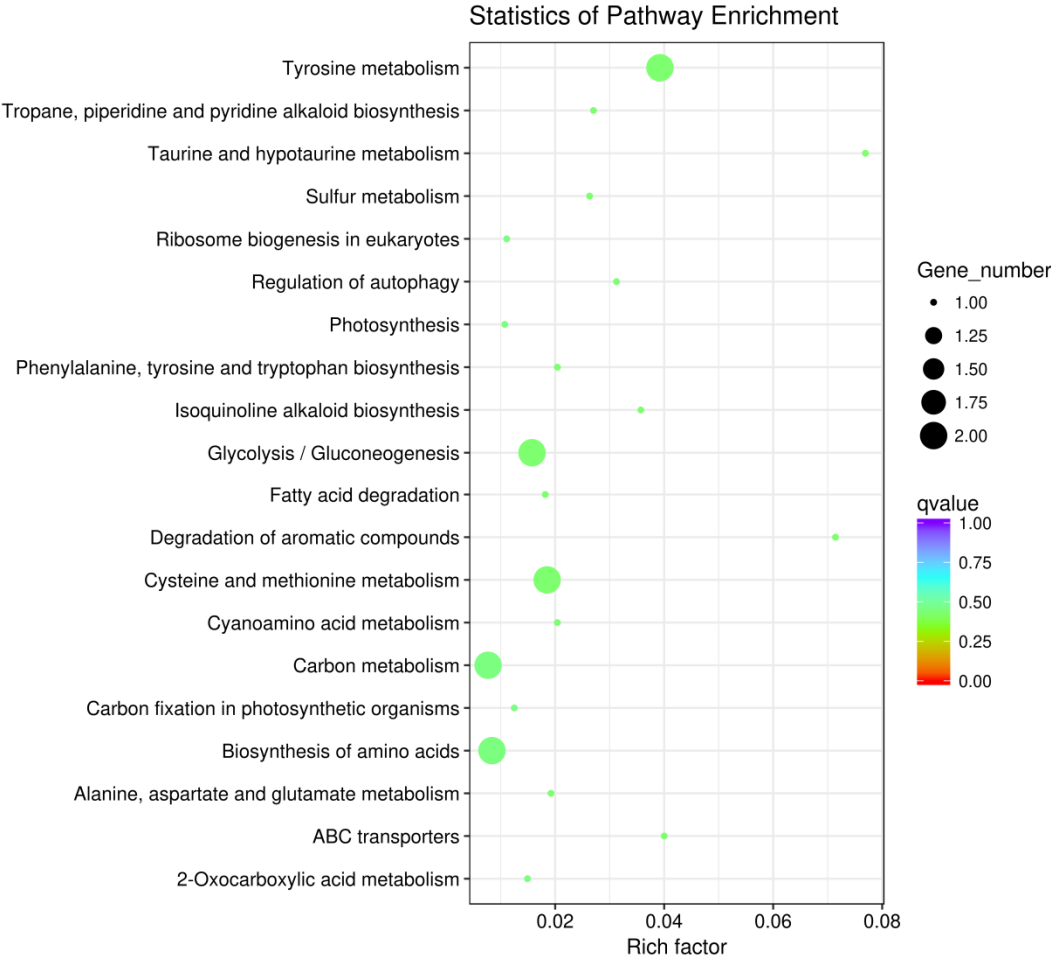

Black module

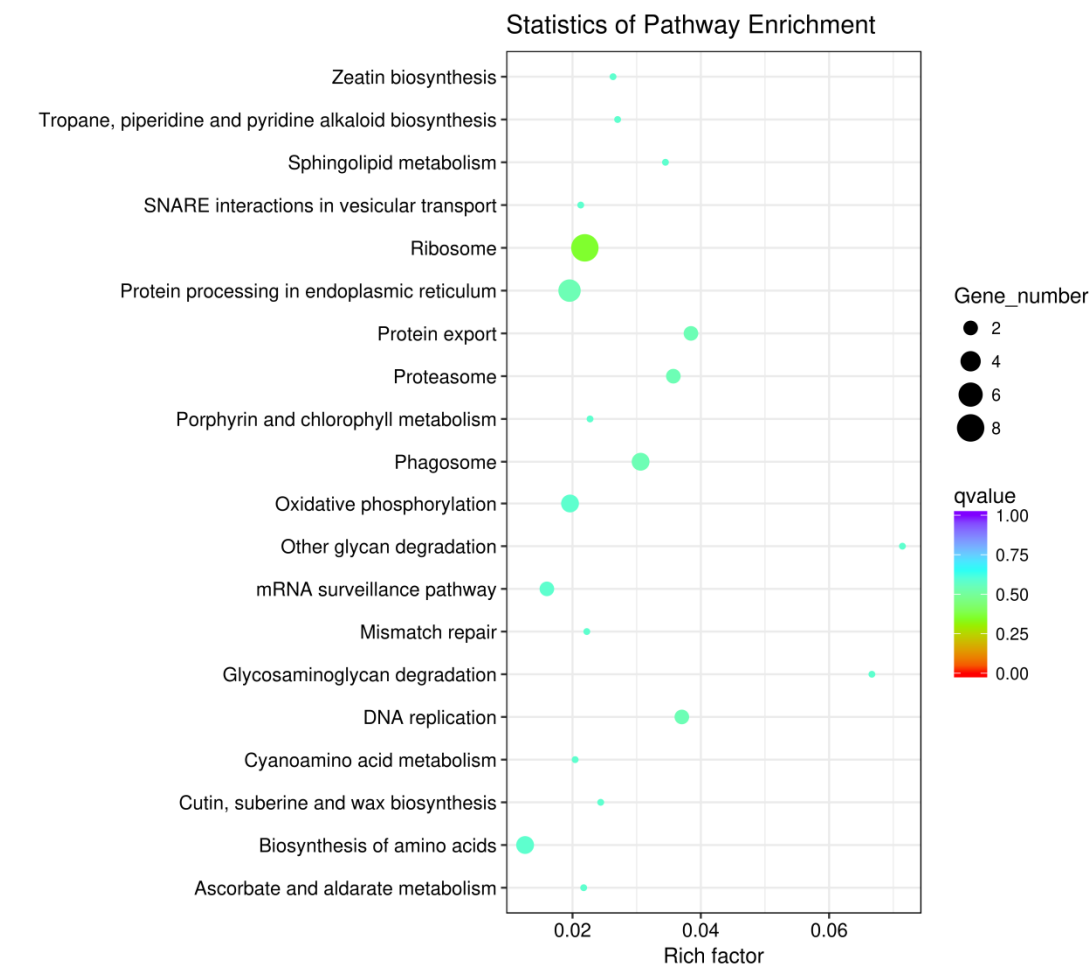

Figure S5. KEGG analysis of DAMs of pink, tan, black module.

Supplement: Supplementary file 1 [file plants-14-01817-s001.zip › Figure S5 KEGG analysis of DEGs of pink, tan, black module..pdf]

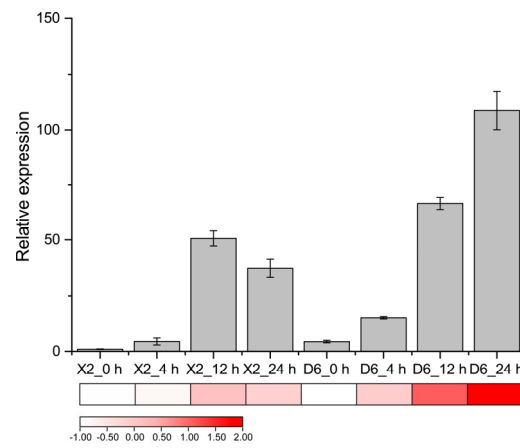

Figure S6 The expression level of laccase gene.

Supplement: Supplementary file 1 [file plants-14-01817-s001.zip › Figure S6 the expression level of laccase gene.pdf]

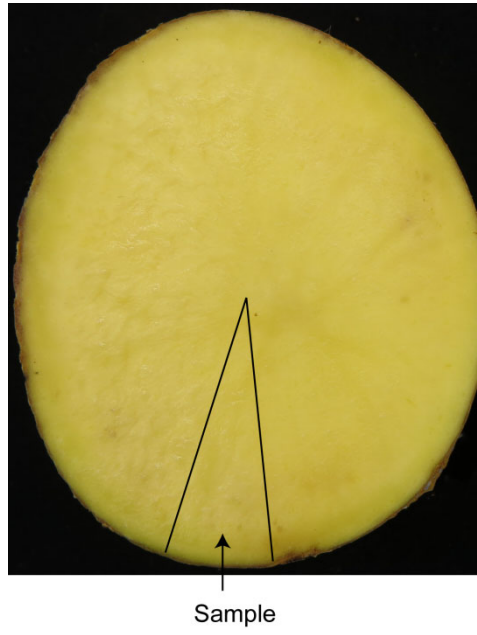

Figure S8. Schematic diagram of RNA-seq sampling of potato tubers

Supplement: Supplementary file 1 [file plants-14-01817-s001.zip › Figure S8 Schematic diagram of RNA-seq sampling of potato tubers.pdf]
